# Electronic Energy Migration in Microtubules

**DOI:** 10.1021/acscentsci.2c01114

**Published:** 2023-01-12

**Authors:** Aarat P. Kalra, Alfy Benny, Sophie M. Travis, Eric A. Zizzi, Austin Morales-Sanchez, Daniel G. Oblinsky, Travis J. A. Craddock, Stuart R. Hameroff, M. Bruce MacIver, Jack A. Tuszyński, Sabine Petry, Roger Penrose, Gregory D. Scholes

**Affiliations:** † Department of Chemistry, New Frick Chemistry Building, Princeton University, Princeton, New Jersey08544, United States; ‡ Department of Molecular Biology, Schultz Laboratory, Princeton University, Princeton, New Jersey08544, United States; § Department of Mechanical and Aerospace Engineering (DIMEAS), Politecnico di Torino, Torino10129, Italy; ∥ Departments of Psychology & Neuroscience, Computer Science, and Clinical Immunology, Nova Southeastern University, Ft. Lauderdale, Florida33314, United States; ⊥ Department of Anesthesiology, Center for Consciousness Studies, University of Arizona, Tucson, Arizona85721, United States; # Department of Anesthesiology, Stanford University School of Medicine, Stanford, California94305, United States; g Department of Physics, University of Alberta, Edmonton, AlbertaT6G 2E1, Canada; h Department of Oncology, University of Alberta, Edmonton, AlbertaT6G 1Z2, Canada; i Mathematical Institute, Andrew Wiles Building, University of Oxford, Radcliffe Observatory Quarter, Woodstock Road, Oxford, OX2 6GG, United Kingdom

## Abstract

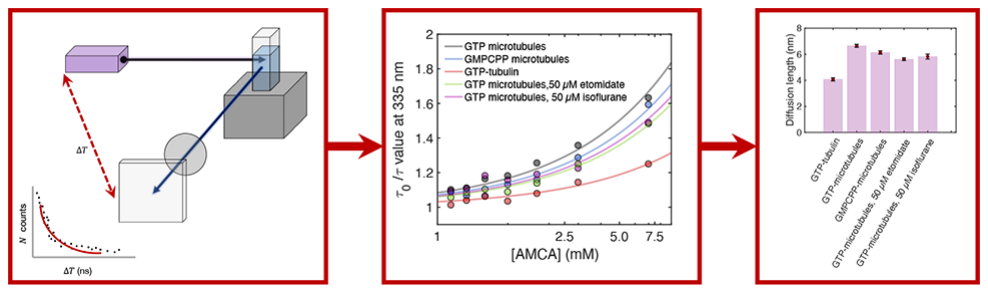

The repeating arrangement of tubulin dimers confers great mechanical strength to microtubules, which are used as scaffolds for intracellular macromolecular transport in cells and exploited in biohybrid devices. The crystalline order in a microtubule, with lattice constants short enough to allow energy transfer between amino acid chromophores, is similar to synthetic structures designed for light harvesting. After photoexcitation, can these amino acid chromophores transfer excitation energy along the microtubule like a natural or artificial light-harvesting system? Here, we use tryptophan autofluorescence lifetimes to probe energy hopping between aromatic residues in tubulin and microtubules. By studying how the quencher concentration alters tryptophan autofluorescence lifetimes, we demonstrate that electronic energy can diffuse over 6.6 nm in microtubules. We discover that while diffusion lengths are influenced by tubulin polymerization state (free tubulin versus tubulin in the microtubule lattice), they are not significantly altered by the average number of protofilaments (13 versus 14). We also demonstrate that the presence of the anesthetics etomidate and isoflurane reduce exciton diffusion. Energy transport as explained by conventional Förster theory (accommodating for interactions between tryptophan and tyrosine residues) does not sufficiently explain our observations. Our studies indicate that microtubules are, unexpectedly, effective light harvesters.

## Abstract 2

In a microtubule, electronic energy can migrate over distances that conventional Förster theory cannot sufficiently explain. Anesthetics etomidate and isoflurane decrease energy migration.

## Introduction

**Figure 1:**
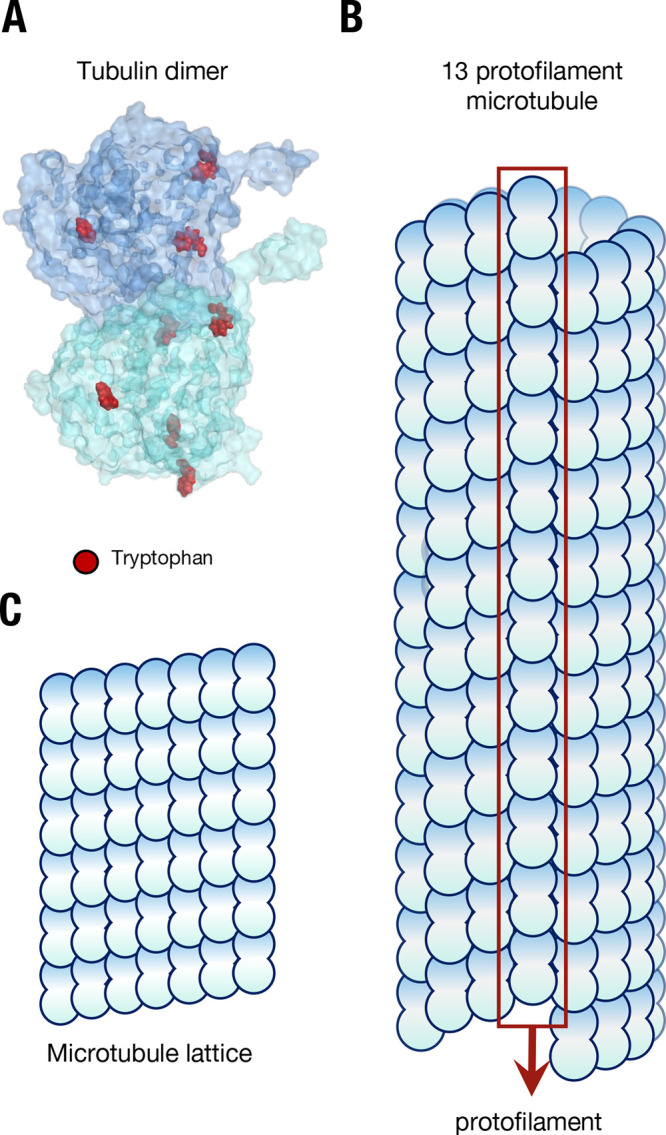
The structure of microtubules from a lattice of tubulin. (A) The tubulin dimer with tryptophan residues marked in red; the C-termini “tails” can be seen protruding from each monomer. (B) The structure of a microtubule, showing constituent arrangement of tubulin dimers, and the presence of a “seam”. (C) The repeating “lattice” of tubulin dimers in a microtubule.

Microtubules are cylindrical polymers of the protein α,β-tubulin that play a variety of structural roles in the cell. Microtubules facilitate chromosome segregation during mitosis, generate intracellular forces, form a “railroad network” for macromolecular transport, and provide mechanical support for organelle positioning.^1,2^ Tubulin is packed in an exquisitely systematic manner in a microtubule, ordered both vertically in columns called protofilaments and horizontally in helical rings (Figure 1A–C^3^). The emergence of a lattice (which, despite the nanometer-scale dimensions of constituent tubulin dimers, can stretch unbroken across several hundreds of micrometers^4^) and the hollow cross section of a microtubule confer structural rigidity, allowing robust execution of a variety of mechanical tasks.^5^ Beyond the cell, these properties have allowed the widespread use of microtubules scaffolds in transport-based nanodevices,^6−10^ wherein motor proteins transport macromolecular cargo across large distances up to the millimeter range by “walking” along the microtubule lattice.^11^

It is well-known that interaromatic residue energy transfer over small distances takes place in proteins.^12−14^ Compared to pigments that absorb light in the visible region of the spectrum, aromatic residues absorb and emit UV light and have low molar extinction coefficients and small photoluminescence quantum yields. Consequently, proteins are not optimal light-harvesting systems for long-range photoexcitation energy migration.^15^ The lattice-type structure of microtubules that incorporates periodic arrays of aromatic amino acids, however, is qualitatively similar to the structure of molecular aggregates^16−19^ and light-harvesting complexes like phycobilisomes,^20,21^ potentially enabling excitation energy transport over long distances.^22^ How long are these distances?

Here, we exploit tryptophan autofluorescence to show that the 2D photoexcitation diffusion length in microtubules (6.6 ± 0.1 nm) is substantially higher than that predicted by conventional Förster theory (1.11 nm for intertyrosine interactions and 1.54 nm including tyrosine-tryptophan interactions). The diffusion length for electronic energy migration in microtubules is thus close to the size of tubulin dimer, comparable to that reported in some photosynthetic complexes.^23−26^ Changing the number of protofilaments from 13 to 14 has a negligible effect on the 2D photoexcitation diffusion length. While a high background signal made it difficult to quantify signal from vinblastine-induced tubulin oligomers, our data also suggest that photoexcitation diffusion lengths decrease in tubulin oligomers (formed in the presence of vinblastine), indicating that photoexcitation may migrate more effectively along protofilaments, as opposed to rings. Notably, we find that the presence of the anesthetics etomidate and isoflurane lowered photoexcitation diffusion coefficients (and thus diffusion lengths) in microtubules. Considering the factors (enumerated above) that make tryptophan a far-from-optimal chromophore, our results demonstrate that electronic energy migration is surprisingly efficient in microtubules.

## Results

### Tryptophan Quenching Rates Can Distinguish between Free GTP-Tubulin and Microtubules

**Figure 2:**
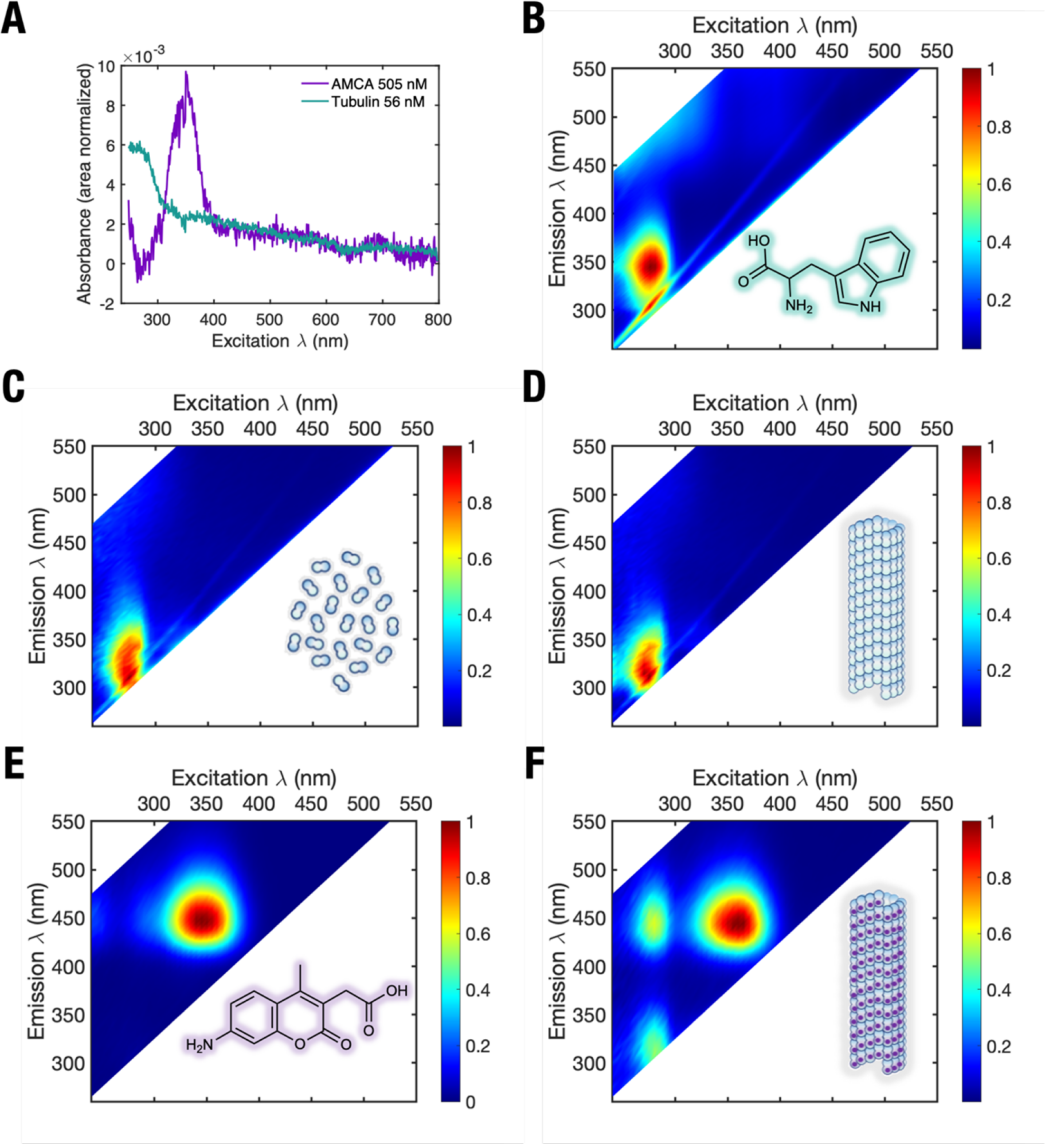
Steady-state spectra of tubulin and microtubules. (A) Absorbance spectra of free tubulin (teal) and free AMCA (purple) in solution. Intensity normalized fluorescence spectra of (B), free DL tryptophan in solution showing highest fluorescence emission at excitation wavelengths between 270 and 300 nm. (C) Unpolymerized GTP-tubulin, (D) microtubules polymerized using GTP-tubulin, (E) free AMCA, and (F) microtubules polymerized using AMCA-labeled GTP-tubulin. An energy transfer peak not observed in Figure 1A,B (at excitation 280–300 nm, and emission at 420–450 nm) is clearly visible. Colors represent photoluminescence intensity.

**Figure 3:**
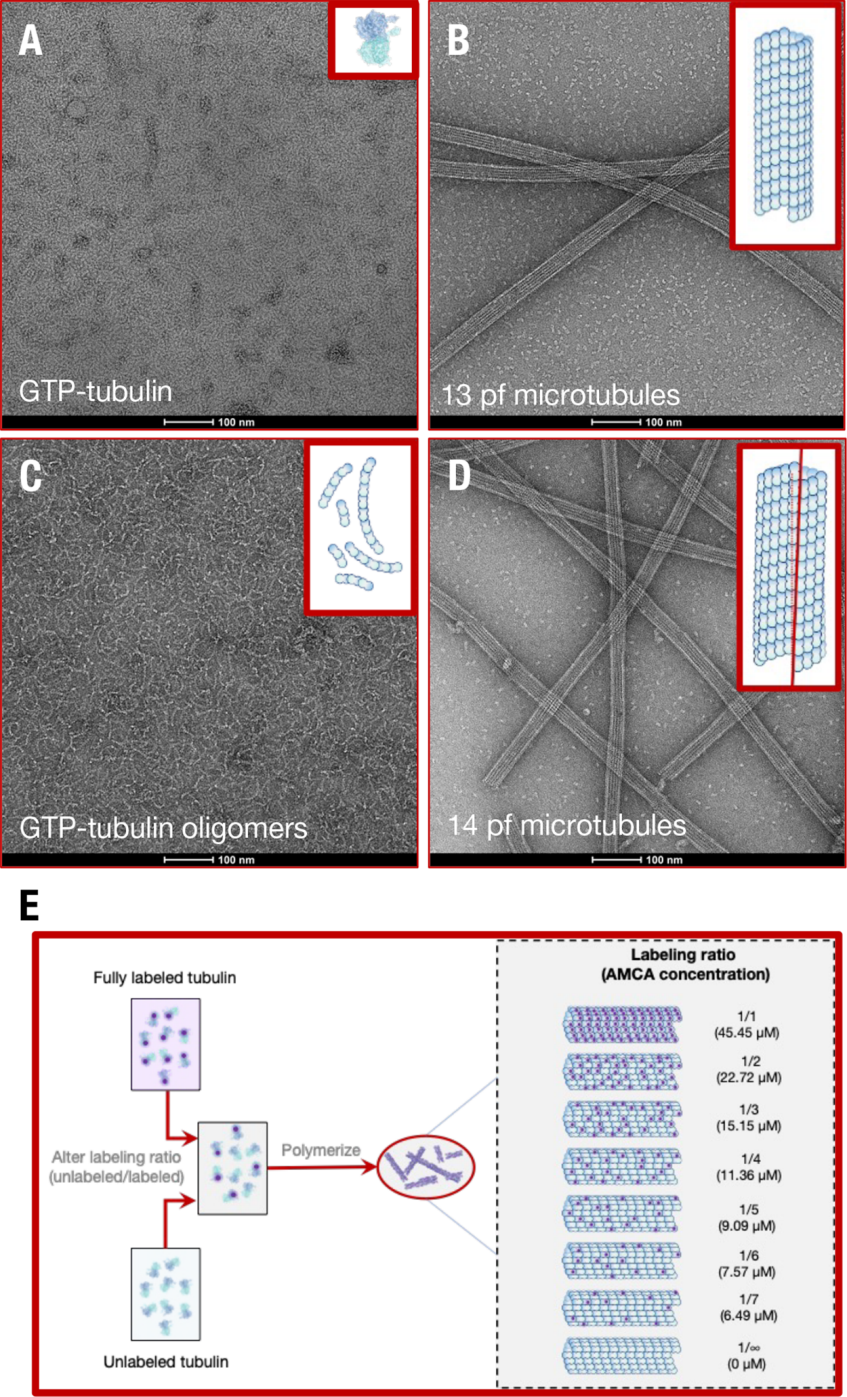
Confirmation and polymerization of tubulin polymorphs. Negative stain electron microscopy of tubulin polymorphic geometries for (A) free GTP-tubulin in solution, (B) GTP-tubulin polymerized 13 protofilament microtubules, (C) free GTP-tubulin oligomers polymerized using 100 μM vinblastine in solution, and (D) GMPCPP-tubulin polymerized 14 protofilament microtubules. Insets show schematics of tubulin polymer structures. Methodology used to perform negative staining is described in SI Appendix. Due to MAPs and drugs that induce microtubule bundling being absent in our solutions and the highly charged C-termini tail of tubulin (expected to cause intermicrotubule repulsion), intermicrotubule separation distances were large enough in solution for electronic energy transfer to be insignificant. (E) Routine to polymerize microtubules with different AMCA labeled tubulin: unlabeled tubulin ratios, thus different AMCA concentrations. See SI Appendix for protocols used to assemble different tubulin polymerization states and perform electron microscopy.

**Figure 4:**
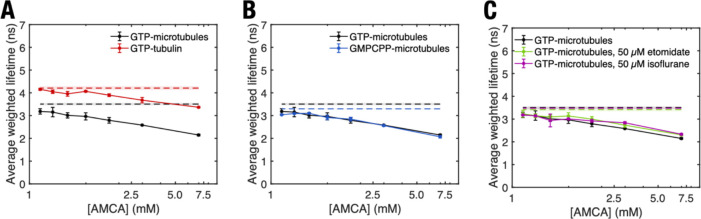
Average weighted tryptophan fluorescence lifetime of different tubulin polymerization states as a function of AMCA concentration. (A) Free GTP tubulin compared to that of microtubules polymerized using GTP-tubulin, (B), microtubules polymerized using GTP-tubulin to that of those polymerized using GMPCPP-tubulin, (C) microtubules polymerized using GTP-tubulin in the presence and absence of etomidate and isoflurane. Dashed lines and shaded regions represent mean and standard deviation of tryptophan lifetimes in different tubulin polymerization states in the absence of AMCA. *p*-Values were calculated to determine significance of differences in average weighted lifetimes between GTP microtubules and other tubulin polymerization states (see Figure S10). Error bars represent standard deviation of experiments conducted *n* = 3 to *n* = 5 times.

**Figure 5:**
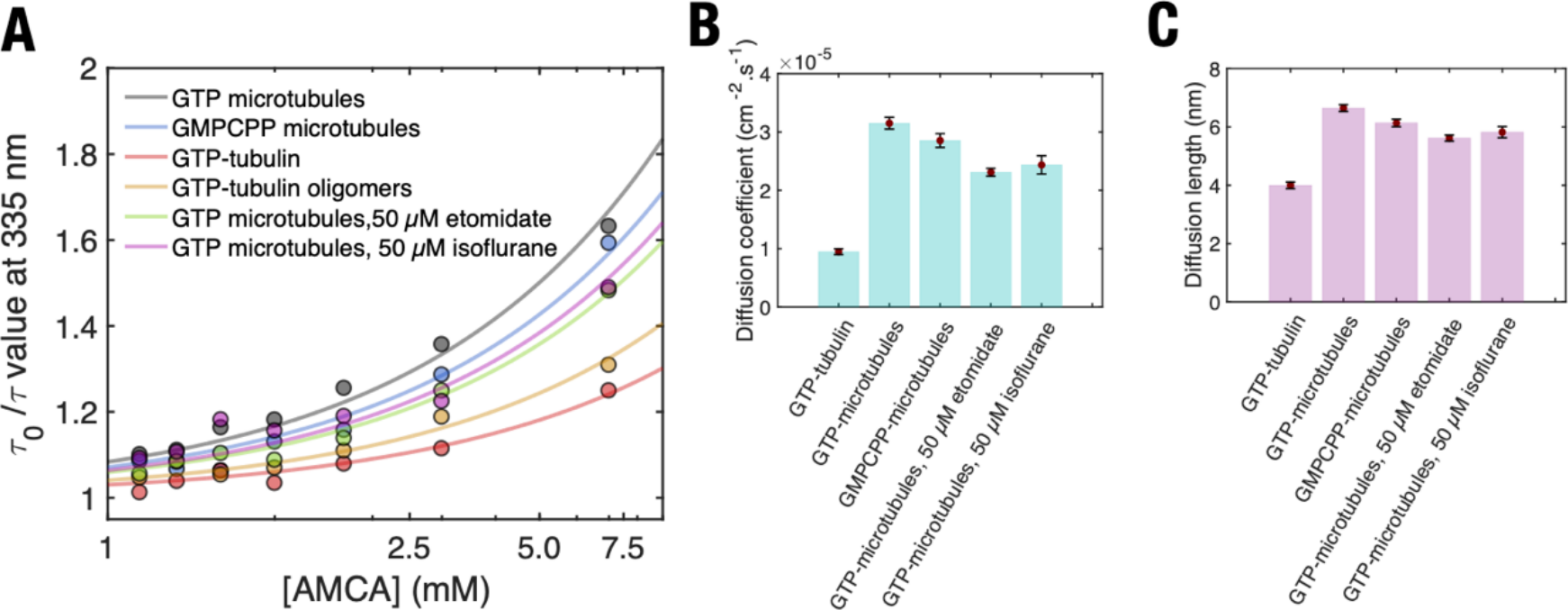
Parameters extracted from Stern–Volmer analysis to estimate extent of diffusion . (A) Stern–Volmer plot for fitting different tubulin polymers with the static quenching model as shown in main text. Lines represent line of best fit to data points shown in the scatterplot. Note the use of a logarithmic scale for the *x*-axis. (B) Diffusion coefficients and (C) diffusion lengths of tryptophan excitation in different tubulin polymerization states. Data are shown in Table S5. The errors associated with (B) are standard errors for the fit to determine the diffusion coefficient. The error bars associated with (C) are calculated after propagating errors from experimental values included in eqs 1 and 2.

We introduced an externally conjugated tryptophan fluorescence quencher, 7-amino-4-methyl coumarin-3-acetic acid (AMCA, Figure 2A) to study intertryptophan hopping in microtubules (SI Appendix, Figure 3E). We found that tryptophan fluorescence lifetimes in microtubules shortened on increasing AMCA concentration (Figure 4A) and, consistent with fluorescence quenching through electronic energy transfer, also observed AMCA fluorescence emission on tryptophan photoexcitation in steady-state experiments (Figure 2E,F). To quantify energy transfer between tryptophan and AMCA, we used the Stern–Volmer equation for static quenching, () where τ is the tryptophan lifetime at AMCA concentration [Q], τ_0_ is the tryptophan lifetime in the absence of AMCA, and *k*_Q_ is the rate constant for quenching by electronic energy transfer (Figure 5A). Our analysis for microtubules and free GTP-tubulin in solution revealed quenching constants of 23.8 ± 0.8 ns^–1^ and 7.5 ± 0.4 ps^–1^, respectively (Figure 4A, Table 1). AMCA itself contributed a negligible signal over the relevant wavelength range (Figure S6). The higher quenching rate in microtubules can be explained by sensitization of AMCA quenching sites remote from the photoexcited tubulin dimer, which can occur by multiple energy transfer steps (as energy migrates from tryptophan to other aromatic residues in its vicinity).

**Table 1:** *K*_Q_ (Mean ± Standard Error of Fit) and Adjusted *R*^2^ Values for Fitting Different Tubulin Polymers with the Static Quenching Model As Shown in the Main Text

tubulin polymerization state	*K*_Q_ (ns^–1^)	Adj *R*^2^
Free GTP-tubulin	7.46 ± 0.4	0.96
13 protofilament microtubules (GTP-tubulin polymerized)	23.84 ± 0.8	0.98
14 protofilament microtubules (GMPCPP-tubulin polymerized)	21.59 ± 0.9	0.98
13 protofilament microtubules with 50 μM etomidate	17.47 ± 0.5	0.99
13 protofilament microtubules with 50 μM isoflurane	18.43 ± 1.2	0.91

To analyze our experimental results, we estimated photoexcitation diffusion lengths using Stern–Volmer analysis.^27^ The measured lifetimes τ and τ_0_ in the Stern–Volmer equation are related to the diffusion coefficient *D* as shown in eq 1:
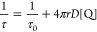
1

Here, *r* is the sum of the tryptophan and AMCA radii (assumed here to be 1 nm). The diffusion coefficient can be used to calculate the photoexcitation diffusion length *L* over the time duration τ to which a photoexcitation migrates on a microtubule before being quenched by AMCA as shown in eq 2.
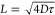
2

Our data yielded a 2D diffusion coefficient of 3.15 ± 0.1 × 10^–5^ cm^2^·s^–1^ and a corresponding diffusion length of 6.64 ± 0.1 nm for microtubules (Table 1, Figure 5B,C). Given that a single tubulin dimer occupies the same volume as a sphere of diameter 7.4 nm (from its dimensions of 4 nm × 6.5 nm × 8 nm), a diffusion length of 6.64 nm suggests that energy transfer between tryptophan residues across a single tubulin dimer can occur in a microtubule. Depending on the locations of the residue where the initial photoexcitation occurred, it could reasonably migrate to the adjacent tubulin dimer within the microtubule lattice.

**Figure 6:**
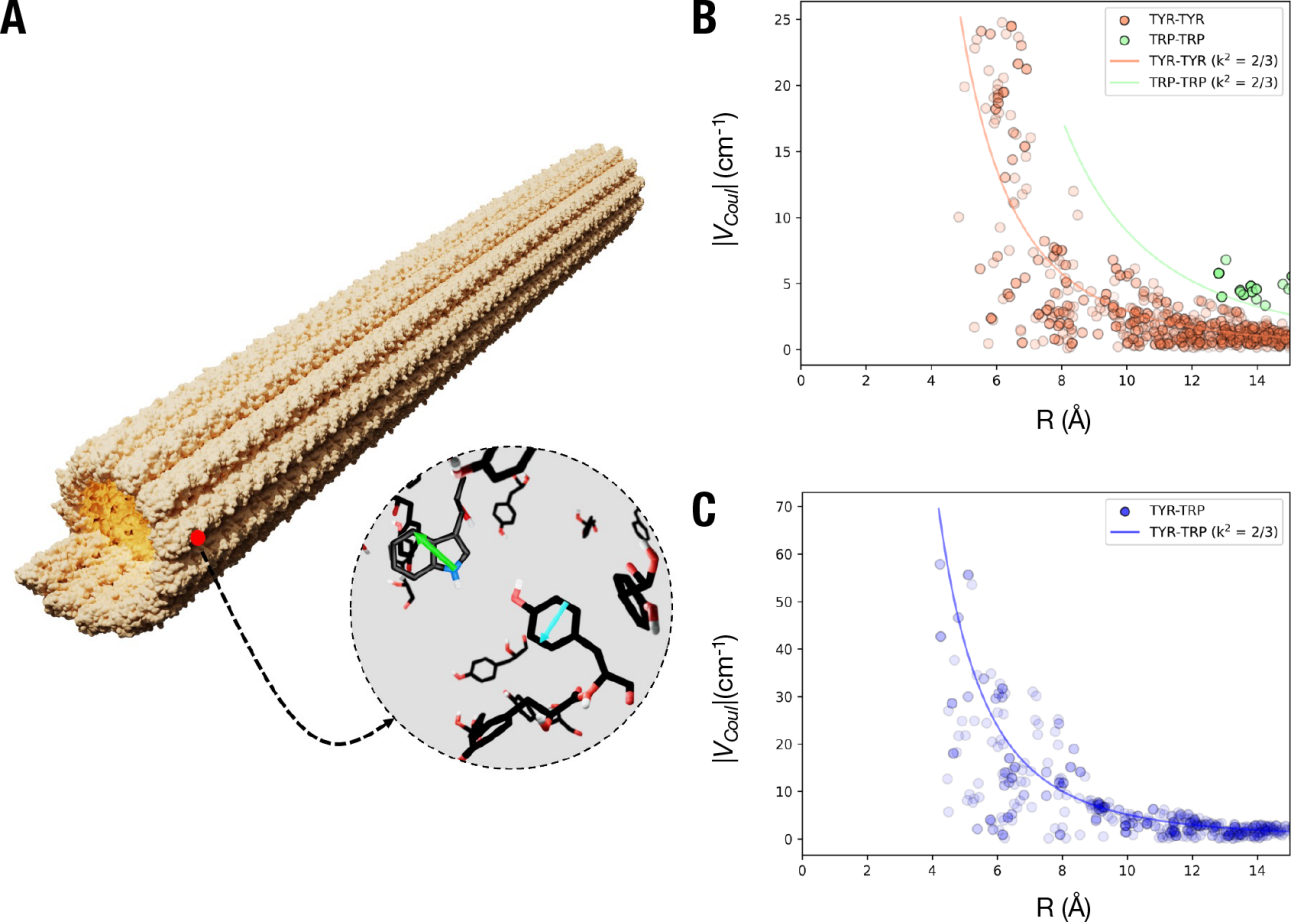
Theoretical estimation of interactions among tryptophan and tyrosine residues. (A) Crystal structure of a microtubule composed of 31 tubulin dimers stacked vertically showing the dipole moment orientations of representative tryptophan (green; TRP) and tyrosine (cyan; TYR) residues. (B) Distribution of the coupling constant *V*_Coul_ between TYR-TYR, TRP-TRP and (C), TYR-TRP residues in 31-dimer long microtubule crystal structure. A projection of *V*_Coul_ with orientation factor (κ^2^) of 2/3 is represented as solid lines.

We simulated (incoherent) energy migration in microtubules using a computational microtubule model composed of 31 stacked tubulin rings (SI Appendix, Figure 6A). Because of its spectral overlap with tryptophan,^12^ tyrosine could serve as an activated intermediate. While the spectral overlap for tryptophan to tyrosine (uphill) energy transfer is small and tryptophan has a higher transition dipole moment (*μ*_TYR_ = 1.18 *D*, *μ*_TRP_ = 2.07 *D*; SI Appendix), the large number of tyrosine residues compared to tryptophan (34 tyrosine residues versus 8 tryptophan residues in the tubulin structure 1JFF^28^) raises the possibility of significant energy transfer between them. Thus, we accounted for both tyrosine and tryptophan chromophores in the simulations.

We calculated the coupling strengths for *homo* (between same residues) and *hetero* (between tyrosine and tryptophan) energy transfer using the ideal dipole approximation:^29^
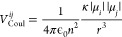
3

Here, *μ*_*i*_ and *μ*_*j*_ denote the transition dipole moments of the *i*th and *j*th residues, κ is the orientation factor, *r* is the separation distance, ϵ_0_ is the permittivity of free space, and *n* is the refractive index of the medium surrounding the microtubule (assumed to be *n* = 1.4). The heteromolecular coupling between tyrosine and tryptophan residues shows the highest contribution to the *V*_Coul_, followed by tyrosine-tyrosine coupling strengths. Tryptophan-tryptophan coupling strengths are the lowest, as a result of longer intermolecular distances between them (Figure 6B,C).

We calculated the diffusion lengths on the microtubule model using a kinetic Monte Carlo algorithm (SI Appendix, Figure S14). The FRET rate (*k*_FRET_) between residues *i* and *j* was calculated using eq 4:^30^
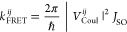
4

Absorbance and emission spectra accommodating the tryptophan peak wavelength to our experimental observations were used to calculate the spectral overlap (*J*_SO_)^31^ by
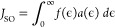
5

Here, *f*(ϵ) and *a*(ϵ) are the emission and absorption spectra of the donor and acceptor residues, respectively, which are area normalized on an energy scale (SI Appendix, Table S11).

We simulated 3000 trajectories, first considering only intertyrosine interactions and then considering tyrosine-tryptophan interactions. In both simulations, the obtained diffusion lengths (1.11 and 1.54 nm, respectively) are appreciably lower than the diffusion lengths obtained experimentally. A possible explanation could be that some of the interchromophore separations are too close for the dipole approximation to be accurate. It is even possible that short-range contributions to the electronic coupling arising from intermolecular orbital overlap may be significant, as has been found to be the case in other tightly packed aggregates.^32−34^ To obtain a rough estimate of the extent of short-range effects, we calculated the hole (*t*_h_) and electron (*t*_e_) transfer Integrals for five representative TYR dimers present in close proximity (interaromatic distance (*R*_IA_) < 6.3 Å) (Table S12, Figure S15, Supporting Information).^35^ We observed charge transfer integral magnitudes ranging up to 419 cm^–1^, which suggests that the intermolecular orbital overlap mediated interactions could increase the electronic coupling and therefore the predicted exciton diffusion length.

### Electronic Energy Migration Is Dampened by Anesthetics Etomidate and Isoflurane

While microtubules have been hypothesized to support long-range dipole-switching of aromatic residues for information processing roles in neurons,^36,37^ experimental support has not yet been provided. Anesthetics have been modeled to bind to tubulin in the microtubule interfering with such long-range interactions, and thereby inhibiting dipole-based information processing.^38−40^ An experimental evaluation of anesthetic action on long-range dipole switching in microtubules has also not yet been performed.

To investigate the influence of anesthetics on tryptophan fluorescence quenching, we introduced the anesthetics etomidate and isoflurane^41,42^ into our assay and measured their effect on tryptophan fluorescence lifetimes. Introducing 50 μM etomidate and isoflurane significantly lowered tryptophan quenching by AMCA in GTP-polymerized microtubules (Figure 4C; Figure S16). The presence of etomidate and isoflurane decreased the 2D diffusion lengths from 6.6 ± 0.1 nm to 5.6 ± 0.1 nm and 5.8 ± 0.2 nm, respectively (Figure 5C). To further investigate the possible binding between etomidate and isoflurane to tubulin in the MT lattice, we built and refined computational models of human tubulin dimers and performed docking simulations (SI Appendix, Figure S11).

Our results showed that both macromolecules exhibited affinities to 16 distinct binding sites (including the vinca, taxol, and colchicine sites), but with low predicted binding energies, which indicates weak to moderate interactions with tubulin, with no distinct preference for any of these sites. Taken together, these findings suggest that etomidate and isoflurane “dampen” energy transfer between tryptophan and AMCA due to changes in the dielectric screening of electronic couplings.

Notably, these anesthetics did not contribute to overall solution fluorescence nor the fluorescence spectra of free GTP-tubulin or AMCA (Figure S6), demonstrating that they did not chemically interfere with either tryptophan or AMCA lifetime.

### Long-Range Electronic Energy Migration Also Occurs in 14 Protofilament Microtubules

Tubulin polymerization in the presence of GMPCPP, a slowly hydrolyzable analog of GTP,^43^ results in the formation of a predominantly 14 protofilament microtubule with different distances between aromatic residues within each tubulin dimer, as well as between neighboring dimers^44^ compared to 13 protofilament microtubules (Figure 3B; Table S1, Table S2). We asked if energy migration would be significantly altered in 14 protofilament microtubules as a consequence. Our results showed that while unlabeled tryptophan fluorescence lifetimes in GMPCPP-microtubules were significantly lower than those in GTP-microtubules (*p*-values < 0.005; Figure S10B), the lifetime trend in the presence of AMCA in GMPCPP-microtubules resembled that of GTP-microtubules (*p*-values > 0.005; Figure 4B). Stern–Volmer analysis revealed different tryptophan quenching rates in GMPCPP-microtubules, which we ascribe to differences in tryptophan fluorescence lifetime in the absence of AMCA. Consequently, long-range electronic energy transfer was observed in GMPCPP-microtubules (diffusion lengths of 6.1 ± 0.1 nm; Figure 5B,C), consistent with multiple step hopping.

## Discussion and Conclusion

While the mechanical properties of microtubules and their interaction with molecular machines have been used as key components in transport-based nanodevices,^6^ their autofluorescence has not yet been exploited. In this work, we find that microtubules are more effective light-harvesters than anticipated for a protein devoid of bound chromophores.

We showed that tryptophan autofluorescence can distinguish between polymerized and unpolymerized tubulin. Stern–Volmer analysis revealed that the 2D diffusion length of tryptophan photoexcitation in microtubules is comparable to that observed in some photosynthetic complexes^23−25^ and organic photovoltaic devices.^45−47^ Our observations suggest that photoexcitation diffusion takes place in a manner that is dependent on the tubulin polymorph type (tubulin dimers versus oligomers versus microtubules). Polymerizing into microtubules enhances diffusion lengths from 4 ± 0.1 nm in free GTP tubulin to 6.64 ± 0.1 nm in microtubules. Subsequently adding tubulin-binding agents (such as anesthetics) decreases the observed diffusion length. The finding of long-range electronic energy transfer shows the versatility of tubulin-based polymers. Of course, it is unlikely that biological systems utilize the light-harvesting properties of tubulin, but it is fascinating that this protein macrostructure exhibits such photoexcitation diffusion lengths.

Photoexcitation diffusion in molecular systems is widely studied by a variety of techniques, often relying on exciton–exciton annihilation.^17,20,21,25,27,48−52^ Like many other fluorophores, tryptophan has a multiexponential fluorescence lifetime decay.^53−55^ Multiexponential fluorescence decays are also observed when electronic energy transfer takes place in a population of fluorophores in proteins (where a fraction of molecules may not undergo electronic energy transfer and may be present in different environments).^56−58^ Here, we take these possibilities together, analyzing the average weighted tryptophan fluorescence lifetime. Therefore, the diffusion length calculated using the method employed in this work is not definite but should be a reliable order of magnitude estimate. The two-dimensional tryptophan photoexcitation diffusion length of 6.64 nm is surprisingly high in microtubules, given that they are optimized to play structural roles in the cell and that the equivalent value for chlorophyll *a*, which is optimized for electronic energy transfer, is only 20–80 nm.^23−25,59^ Because tryptophan absorbs light poorly (molar extinction coefficient of ∼5600 M^–1^ cm^–1^^60^) compared to chlorophyll *a* (molar extinction coefficient of ∼110,000 M^–1^ cm^–1^^61^) photoexcitation diffusion on a lengths scale of several nanometers was not expected.

We also attempted to understand the preferred path of photoexcitation migration. It is likely that photoexcitation energy migration takes place preferentially along a protofilament or along the tubulin ring along a helical path (Figure S13). To determine if long-range photoexcitation migration could take place in the absence of lateral contacts between tubulin dimers, we polymerized GTP-tubulin in the presence of 100 μM vinblastine, which binds at the axial interface between two tubulin dimers, forming tubulin oligomers (Figure 3C).^62^ Vinblastine produced a signal in our fluorescence lifetime measurement (Figure S3B), making it difficult to quantify the tryptophan fluorescence because of overlapping fluorescence. Thus, after estimating the time-resolved fluorescence decay of tubulin oligomers, we subtracted the short lifetime component (Figures S8 and S9), using only two lifetimes to determine the diffusion parameters of tryptophan (Figure S7, Figure 5A). Nevertheless, the diffusion length obtained from such analysis (4.6 ± 0.1 nm) indicates long-range electronic energy transport in oligomers. While these diffusion lengths are lower than those observed in microtubules, we speculate that longitudinal contacts between tubulin dimers, resembling those in a protofilament, are sufficient to induce long-range energy transport in tubulin polymers. The diffusion lengths observed in GMPCPP microtubules also support the notion that a repeating arrangement of tubulin dimers is sufficient to allow long-range photoexcitation energy transfer.

It is also worth noting that introducing a convulsant (50 μM picrotoxin) also decreased tryptophan quenching by AMCA. These additional experiments that we do not report here suggest that anesthetics are not the only macromolecules that decrease photoexcitation diffusion in microtubules. Thus, we anticipate that in addition to anesthetics, microtubule associated proteins (MAPs), microtubule associated drugs (MADs), and other tubulin interacting agents form a “cytoexcitonic” framework for altering electronic energy migration in microtubule-based devices. Biochemically tuned electronic energy migration would allow microtubule architectures to be used as targets for photobiomodulation^9,63^ and to transduce photonic energy in biohybrid devices.^64^

A key finding of our study is that photoexcitation diffusion cannot be explained via conventional Förster theory, which accounts for only dipole–dipole interactions between tryptophan and tyrosine residues. A satisfactory explanation of our observations may include solvent screening by the protein matrix,^56^ electronic coupling beyond the point dipole approximation in Förster theory,^65,66^ and accommodation for molecular exciton states in tubulin.^26^ The tuning of photoexcitation diffusion length through biochemical parameters of microtubules (tubulin polymerization, introduction of anesthetics) was a further intriguing observation. Our work shows that protein polymers may be suitable for active materials in biologically sourced electronic devices where UV photoexcitation is desired.

## Methods

### Tubulin Polymerization

Tubulin was polymerized and stabilized as described in previous literature.^10,67^ Briefly, porcine brain tubulin stock was prepared by reconstituting lyophilized tubulin powder (T240; Cytoskeleton Inc., Denver, CO, USA) in BRB80 buffer supplemented with 10% glycerol at 1 mM GTP. AMCA labeled tubulin solution was prepared by reconstituting lyophilized AMCA labeled tubulin powder (TL440m; Cytoskeleton Inc., Denver, CO, USA) in BRB80 supplemented with 10% glycerol and 1 mM GTP, to a final concentration of 45 μM tubulin. Labeling efficiency was 1–2 AMCA molecules per tubulin dimer, attached at random surface lysines. Different volumes of labeled tubulin solution were added to tubulin solution to prepare solutions containing different AMCA labeling ratios. These solutions were incubated for 45 min at 37 °C to polymerize microtubules and subsequently stabilized using 100 μM paclitaxel (BRB80T) to ensure no microtubule depolymerization. Isoflurane and etomidate were added after microtubule polymerization and stabilization using paclitaxel had been performed. All microtubule containing solutions contained excess paclitaxel (a potent microtubule stabilizing agent;^68^ 100 μM paclitaxel used to stabilize 2.2 μM tubulin). The binding of paclitaxel counters the conformational effect of GTP-hydrolysis on microtubules, stabilizing microtubules against depolymerization.^69,70^ Consequently, conformational changes in the tubulin structure within the microtubule lattice in the presence of paclitaxel are not expected. Free GTP-tubulin stock was incubated with 100 μM vinblastine (BRB80V) for 45 min at 37 °C to stabilize GTP-tubulin oligomers. Measurements on free tubulin in solution were performed in the presence of 10 mM CaCl_2_ (BRB80Ca) to ensure that free tubulin remained in solution. Stock solutions of isoflurane, etomidate, and vinblastine were prepared in DMSO.

### Steady-State Measurements

The sample absorbance was measured as a function of light frequency using a UV–vis spectrometer complemented with an integrating sphere (Cary 6000i, Agilent Technologies, Santa Clara, CA, USA). The absorption spectra were recorded using a background consisting of BRB80T (BRB80 supplemented with 1 mM GTP or GMPCPP and 100 μM paclitaxel), for microtubules, and BRB80Ca (BRB80 supplemented with 1 mM GTP or GMPCPP and 1 mM CaCl_2_) for free GTP-tubulin solutions. Steady-state fluorescence spectroscopy was performed using a Photon Technology International (PTI) QuantaMaster 40-F NA spectrofluorometer. All steady-state measurements were performed at room temperature.

### Time-Correlated Single Photon Counting (TCPSC)

The time-resolved fluorescence of each sample was measured using a DeltaFlex TCSPC instrument Horiba (Kyoto, Kyoto, Japan). The input light was provided using a 305 nm LED light source (DD300; Horiba, Kyoto, Kyoto, Japan). The usage of 305 nm light ensured that tyrosine and phenylalanine were not photoexcited. A long pass dielectric filter with a transition wavelength of 325 nm was placed after the solution and before the detector to reduce scatter signal. The fluorescence emission was detected at 335 nm. These excitation and detection wavelengths were selected to ensure that tyrosine and phenylalanine were not photoexcited. The time-resolved spectra were corrected for instrument response function (IRF) using an aqueous Ludox colloidal silica solution (Figure S3a). The measurement range was set from 0 to 100 ns, with the recording set to stop measurements when a maximum of 10,000 counts was attained. Obtained time-resolved fluorescence decays were fit to eq S1:
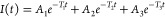
S1

The fit parameters *T*_1_ and *T*_2_ represented tryptophan lifetimes in the protein, while *T*_3_ represented the fit parameter for residual scattering from different tubulin polymers, and *A*_1_, *A*_2_, and *A*_3_ represented the relative contributions of each lifetime (amplitude). Average weighted values were estimated by finding the weighted lifetime of tryptophan fluorescence lifetime (the parameters *T*_1_, *T*_2_, and *T*_3_ were weighted using amplitudes *A*_1_, *A*_2_, and *A*_3_) and then determining the average of this value over *n* = 3 to 5 experiments.

### Negative Stain Electron Microscopy

2 μM tubulin was polymerized as described as above, then either used at 2 μM or diluted immediately prior to grid preparation to 400 nM. Dilution was performed in the appropriate polymerization buffer at room temperature. A 3 μL tubulin mixture was added to glow-discharged CF400-Cu grids (Electron Microscopy Sciences, Hatfield, PA, USA) and stained with 0.75% uranyl formate. Samples were imaged using a Thermo Scientific Talos L120C transmission electron microscope operating at 200 keV. The nominal magnification was 72,000, and defocus values ranged from −0.5 to −2.0 μm. Micrographs were recorded on a Thermo Scientific Ceta-M 4k × 4k-pixel CMOS camera using the TIA data collection software (Thermo Scientific, Waltham, MA, USA), at a calibrated pixel size of 2.02 Å. All TCSPC experiments were performed at room temperature.

## Data Availability

The authors declare that the data supporting the findings of this study are available within the paper and its Supporting Information files, as well as from the corresponding author upon reasonable request.

## Supporting Information Available

Tubulin polymerization, steady-state optical absorption spectroscopy, steady-state fluorescence spectroscopy, time correlated single photon counting (tcpsc), negative stain electron microscopy, ret characteristic length determination, docking of etomidate, isoflurane, and picrotoxin to human tubulin, homology modeling, structure equilibration, site identification and docking, supplementary figures (PDF)

## References

[ref1] Kapitein L. C.; Hoogenraad C. C. Building the Neuronal Microtubule Cytoskeleton. Neuron 2015, 87 (3), 492 10.1016/j.neuron.2015.05.046. 26247859

[ref2] Matis M. The Mechanical Role of Microtubules in Tissue Remodeling. BioEssays 2020, 42 (5), 1900244 10.1002/bies.201900244. 32249455

[ref3] Li H.; DeRosier D. J.; Nicholson W. V.; Nogales E.; Downing K. H. Microtubule Structure at 8Å Resolution. Structure 2002, 10 (10), 1317 10.1016/S0969-2126(02)00827-4. 12377118

[ref4] Schaedel L.; Triclin S.; Chrétien D.; Abrieu A.; Aumeier C.; Gaillard J.; Blanchoin L.; Théry M.; John K. Lattice Defects Induce Microtubule Self-Renewal. Nat. Phys. 2019, 15 (8), 830 10.1038/s41567-019-0542-4. 31867047 PMC6924994

[ref5] Mohrbach H.; Johner A.; Kulić I. M. Cooperative lattice dynamics and anomalous fluctuations of microtubules. Eur. Biophys. J. 2012, 41 (2), 217 10.1007/s00249-011-0778-0. 22173449

[ref6] Saper G.; Hess H. Synthetic Systems Powered by Biological Molecular Motors. Chem. Rev. 2020, 120, 288 10.1021/acs.chemrev.9b00249. 31509383

[ref7] Reuther C.; Catalano R.; Salhotra A.; Vemula V.; Korten T.; Diez S.; Månsson A. Comparison of actin- and microtubule-based motility systems for application in functional nanodevices. New J. Phys. 2021, 23 (7), 075007 10.1088/1367-2630/ac10ce.

[ref8] Korten T.; Månsson A.; Diez S. Towards the application of cytoskeletal motor proteins in molecular detection and diagnostic devices. Curr. Opin. Biotechnol. 2010, 21 (4), 477 10.1016/j.copbio.2010.05.001. 20860918

[ref9] Kalra A. P.; Eakins B. B.; Patel S. D.; Ciniero G.; Rezania V.; Shankar K.; Tuszynski J. A. All Wired Up: An Exploration of the Electrical Properties of Microtubules and Tubulin. ACS Nano 2020, 14 (12), 16301 10.1021/acsnano.0c06945. 33213135

[ref10] Kalra A. P.; Eakins B. B.; Vagin S. I.; Wang H.; Patel S. D.; Winter P.; Aminpour M.; Lewis J. D.; Rezania V.; Shankar K. A Nanometric Probe of the Local Proton Concentration in Microtubule-Based Biophysical Systems. Nano Lett. 2022, 22, 517 10.1021/acs.nanolett.1c04487. 34962401

[ref11] Sweeney H. L.; Holzbaur E. L. F. Motor Proteins. Cold Spring Harbor Perspectives in Biology 2018, 10 (5), a021931 10.1101/cshperspect.a021931. 29716949 PMC5932582

[ref12] Karreman G.; Steele R. H.; Szent-Györgyi A. On Resonance Transfer of Excitation Energy Between Aromatic Amino Acids in Proteins. Proc. Natl. Acad. Sci. U. S. A. 1958, 44 (2), 140 10.1073/pnas.44.2.140. 16590156 PMC335378

[ref13] Bannister T. T. Energy transfer between chromophore and protein in phycocyanin. Arch. Biochem. Biophys. 1954, 49 (1), 222 10.1016/0003-9861(54)90182-4. 13139686

[ref14] Weber G. Fluorescence-polarization spectrum and electronic-energy transfer in proteins. Biochem. J. 1960, 75 (2), 345 10.1042/bj0750345. 13843296 PMC1204431

[ref15] Scholes G. D.; Fleming G. R.; Olaya-Castro A.; van Grondelle R. Lessons from nature about solar light harvesting. Nat. Chem. 2011, 3 (10), 763 10.1038/nchem.1145. 21941248

[ref16] Rehhagen C.; Stolte M.; Herbst S.; Hecht M.; Lochbrunner S.; Würthner F.; Fennel F. Exciton Migration in Multistranded Perylene Bisimide J-Aggregates. J. Phys. Chem. Lett. 2020, 11 (16), 6612 10.1021/acs.jpclett.0c01669. 32686422

[ref17] Caram J. R.; Doria S.; Eisele D. M.; Freyria F. S.; Sinclair T. S.; Rebentrost P.; Lloyd S.; Bawendi M. G. Room-Temperature Micron-Scale Exciton Migration in a Stabilized Emissive Molecular Aggregate. Nano Lett. 2016, 16 (11), 6808 10.1021/acs.nanolett.6b02529. 27689389

[ref18] Haedler A. T.; Kreger K.; Issac A.; Wittmann B.; Kivala M.; Hammer N.; Köhler J.; Schmidt H.-W.; Hildner R. Long-range energy transport in single supramolecular nanofibres at room temperature. Nature 2015, 523 (7559), 196 10.1038/nature14570. 26156373

[ref19] Eisele D. M.; Arias D. H.; Fu X.; Bloemsma E. A.; Steiner C. P.; Jensen R. A.; Rebentrost P.; Eisele H.; Tokmakoff A.; Lloyd S.; Nelson K. A.; Nicastro D.; Knoester J.; Bawendi M. G. Robust excitons inhabit soft supramolecular nanotubes. Proc. Natl. Acad. Sci. U. S. A. 2014, 111 (33), E3367 10.1073/pnas.1408342111. 25092336 PMC4142990

[ref20] Lunt R. R.; Benziger J. B.; Forrest S. R. Relationship between Crystalline Order and Exciton Diffusion Length in Molecular Organic Semiconductors. Adv. Mater. 2010, 22 (11), 1233 10.1002/adma.200902827. 20437510

[ref21] Najafov H.; Lee B.; Zhou Q.; Feldman L. C.; Podzorov V. Observation of long-range exciton diffusion in highly ordered organic semiconductors. Nature materials 2010, 9 (11), 938 10.1038/nmat2872. 20935655

[ref22] Kurian P.; Obisesan T.; Craddock T. J. Oxidative species-induced excitonic transport in tubulin aromatic networks: Potential implications for neurodegenerative disease. Journal of Photochemistry and Photobiology B: Biology 2017, 175, 109 10.1016/j.jphotobiol.2017.08.033. 28865316 PMC5610651

[ref23] Amarnath K.; Bennett D. I. G.; Schneider A. R.; Fleming G. R. Multiscale model of light harvesting by photosystem II in plants. Proc. Natl. Acad. Sci. U. S. A. 2016, 113 (5), 1156 10.1073/pnas.1524999113. 26787911 PMC4747709

[ref24] Bennett D. I. G.; Fleming G. R.; Amarnath K. Energy-dependent quenching adjusts the excitation diffusion length to regulate photosynthetic light harvesting. Proc. Natl. Acad. Sci. U. S. A. 2018, 115 (41), E9523 10.1073/pnas.1806597115. 30237283 PMC6187178

[ref25] Geacintov N. E.; Breton J.; Swenberg C. E.; Paillotin G. A Single Pulse Picosecond Laser Study of Exciton Dynamics in Chloroplasts. Photochem. Photobiol. 1977, 26 (6), 629 10.1111/j.1751-1097.1977.tb07542.x.

[ref26] Mirkovic T.; Ostroumov E. E.; Anna J. M.; van Grondelle R.; Govindjee; Scholes G. D. Light Absorption and Energy Transfer in the Antenna Complexes of Photosynthetic Organisms. Chem. Rev. 2017, 117 (2), 249 10.1021/acs.chemrev.6b00002. 27428615

[ref27] Lin J. D. A.; Mikhnenko O. V.; Chen J.; Masri Z.; Ruseckas A.; Mikhailovsky A.; Raab R. P.; Liu J.; Blom P. W. M.; Loi M. A.; et al. Systematic study of exciton diffusion length in organic semiconductors by six experimental methods. Materials Horizons 2014, 1 (2), 280 10.1039/C3MH00089C.

[ref28] Löwe J.; Li H.; Downing K. H.; Nogales E. Refined Structure of αβ-tubulin at 3.5 Å Resolution. J. Mol. Biol. 2001, 313 (5), 1045 10.1006/jmbi.2001.5077. 11700061

[ref29] Krueger B. P.; Scholes G. D.; Fleming G. R. Calculation of Couplings and Energy-Transfer Pathways between the Pigments of LH2 by the ab Initio Transition Density Cube Method. J. Phys. Chem. B 1998, 102 (27), 5378 10.1021/jp9811171.

[ref30] Scholes G. D.; Fleming G. R. On the Mechanism of Light Harvesting in Photosynthetic Purple Bacteria: B800 to B850 Energy Transfer. J. Phys. Chem. B 2000, 104 (8), 1854 10.1021/jp993435l.

[ref31] Taniguchi M.; Du H.; Lindsey J. S. PhotochemCAD 3: Diverse Modules for Photophysical Calculations with Multiple Spectral Databases. Photochem. Photobiol. 2018, 94 (2), 277 10.1111/php.12862. 29166541

[ref32] Scholes G. D.; Ghiggino K. P.; Oliver A. M.; Paddon-Row M. N. Intramolecular electronic energy transfer between rigidly linked naphthalene and anthracene chromophores. J. Phys. Chem. 1993, 97 (46), 11871 10.1021/j100148a006.

[ref33] Hestand N. J.; Spano F. C. Expanded Theory of H- and J-Molecular Aggregates: The Effects of Vibronic Coupling and Intermolecular Charge Transfer. Chem. Rev. 2018, 118 (15), 7069 10.1021/acs.chemrev.7b00581. 29664617

[ref34] Scholes G. D.; Ghiggino K. P.; Oliver A. M.; Paddon-Row M. N. Through-space and through-bond effects on exciton interactions in rigidly linked dinaphthyl molecules. J. Am. Chem. Soc. 1993, 115 (10), 4345 10.1021/ja00063a061.

[ref35] Baumeier B.; Kirkpatrick J.; Andrienko D. Density-functional based determination of intermolecular charge transfer properties for large-scale morphologies. Phys. Chem. Chem. Phys. 2010, 12 (36), 11103 10.1039/c002337j. 20689881

[ref36] Hameroff S.; Penrose R. Orchestrated reduction of quantum coherence in brain microtubules: A model for consciousness. Mathematics and Computers in Simulation 1996, 40 (3), 453 10.1016/0378-4754(96)80476-9.

[ref37] Hameroff S.; Penrose R. Consciousness in the universe: A review of the ‘Orch OR’ theory. Physics of Life Reviews 2014, 11 (1), 39 10.1016/j.plrev.2013.08.002. 24070914

[ref38] Craddock T. J. A.; St. George M.; Freedman H.; Barakat K. H.; Damaraju S.; Hameroff S.; Tuszynski J. A. Computational predictions of volatile anesthetic interactions with the microtubule cytoskeleton: Implications for side effects of general anesthesia. PLoS One 2012, 7 (6), e37251 10.1371/journal.pone.0037251. 22761654 PMC3382613

[ref39] Craddock T. J.; Kurian P.; Preto J.; Sahu K.; Hameroff S. R.; Klobukowski M.; Tuszynski J. A. Anesthetic alterations of collective terahertz oscillations in tubulin correlate with clinical potency: Implications for anesthetic action and post-operative cognitive dysfunction. Sci. Rep. 2017, 7 (1), 1 10.1038/s41598-017-09992-7. 28852014 PMC5575257

[ref40] Craddock T. J. A.; Friesen D.; Mane J.; Hameroff S.; Tuszynski J. A. The feasibility of coherent energy transfer in microtubules. J. R. Soc., Interface 2014, 11 (100), 20140677 10.1098/rsif.2014.0677. 25232047 PMC4191094

[ref41] Forman S. A.; Warner D. S. Clinical and Molecular Pharmacology of Etomidate. Anesthesiology 2011, 114 (3), 695 10.1097/ALN.0b013e3181ff72b5. 21263301 PMC3108152

[ref42] Constantinides C.; Murphy K. Molecular and Integrative Physiological Effects of Isoflurane Anesthesia: The Paradigm of Cardiovascular Studies in Rodents using Magnetic Resonance Imaging. Frontiers in Cardiovascular Medicine 2016, 3. 10.3389/fcvm.2016.00023 PMC4965459 27525256

[ref43] Hyman A. A.; Salser S.; Drechsel D.; Unwin N.; Mitchison T. J. Role of GTP Hydrolysis in Microtubule Dynamics: Information from a Slowly Hydrolyzable Analogue, GMPCPP. Molecular biology of the cell 1992, 3 (10), 1155 10.1091/mbc.3.10.1155. 1421572 PMC275679

[ref44] Zhang R.; LaFrance B.; Nogales E. Separating the effects of nucleotide and EB binding on microtubule structure. Proc. Natl. Acad. Sci. U. S. A. 2018, 115 (27), E6191 10.1073/pnas.1802637115. 29915050 PMC6142192

[ref45] Penwell S. B.; Ginsberg L. D. S.; Noriega R.; Ginsberg N. S. Resolving ultrafast exciton migration in organic solids at the nanoscale. Nat. Mater. 2017, 16 (11), 1136 10.1038/nmat4975. 28920937

[ref46] Markov D. E.; Amsterdam E.; Blom P. W. M.; Sieval A. B.; Hummelen J. C. Accurate Measurement of the Exciton Diffusion Length in a Conjugated Polymer Using a Heterostructure with a Side-Chain Cross-Linked Fullerene Layer. J. Phys. Chem. A 2005, 109 (24), 5266 10.1021/jp0509663. 16839049

[ref47] Mikhnenko O. V.; Blom P. W. M.; Nguyen T.-Q. Exciton diffusion in organic semiconductors. Energy Environ. Sci. 2015, 8 (7), 1867 10.1039/C5EE00925A.

[ref48] Malý P.; Lüttig J.; Turkin A.; Dostál J.; Lambert C.; Brixner T. From wavelike to sub-diffusive motion: exciton dynamics and interaction in squaraine copolymers of varying length. Chemical Science 2020, 11 (2), 456 10.1039/C9SC04367E. PMC8146531 34084345

[ref49] Kriete B.; Lüttig J.; Kunsel T.; Malý P.; Jansen T. L. C.; Knoester J.; Brixner T.; Pshenichnikov M. S. Interplay between structural hierarchy and exciton diffusion in artificial light harvesting. Nat. Commun. 2019, 10 (1), 4615 10.1038/s41467-019-12345-9. 31601795 PMC6787233

[ref50] Mikhnenko O. V.; Kuik M.; Lin J.; van der Kaap N.; Nguyen T.-Q.; Blom P. W. M. Trap-Limited Exciton Diffusion in Organic Semiconductors. Adv. Mater. 2014, 26 (12), 1912 10.1002/adma.201304162. 24804328

[ref51] Bressan G.; Jirasek M.; Anderson H. L.; Heisler I. A.; Meech S. R. Exciton-Exciton Annihilation as a Probe of Exciton Diffusion in Large Porphyrin Nanorings. J. Phys. Chem. C 2020, 124 (34), 18416 10.1021/acs.jpcc.0c04546.

[ref52] Menke S. M.; Luhman W. A.; Holmes R. J. Tailored exciton diffusion in organic photovoltaic cells for enhanced power conversion efficiency. Nat. Mater. 2013, 12 (2), 152 10.1038/nmat3467. 23142837

[ref53] Ghisaidoobe A. B.; Chung S. J. Intrinsic tryptophan fluorescence in the detection and analysis of proteins: a focus on Förster resonance energy transfer techniques. International journal of molecular sciences 2014, 15 (12), 22518 10.3390/ijms151222518. 25490136 PMC4284722

[ref54] Albani J. Origin of tryptophan fluorescence lifetimes part 1. Fluorescence lifetimes origin of tryptophan free in solution. J. Fluoresc. 2014, 24 (1), 93 10.1007/s10895-013-1277-8. 23912963

[ref55] Albani J. Origin of tryptophan fluorescence lifetimes. Part 2: Fluorescence lifetimes origin of tryptophan in proteins. J. Fluoresc. 2014, 24 (1), 105 10.1007/s10895-013-1274-y. 23907253

[ref56] Curutchet C.; Kongsted J.; Muñoz-Losa A.; Hossein-Nejad H.; Scholes G. D.; Mennucci B. Photosynthetic Light-Harvesting Is Tuned by the Heterogeneous Polarizable Environment of the Protein. J. Am. Chem. Soc. 2011, 133 (9), 3078 10.1021/ja110053y. 21322565

[ref57] Scholes G. D.; Curutchet C.; Mennucci B.; Cammi R.; Tomasi J. How Solvent Controls Electronic Energy Transfer and Light Harvesting. J. Phys. Chem. B 2007, 111 (25), 6978 10.1021/jp072540p. 17550286

[ref58] Petrich J.; Chang M.; McDonald D.; Fleming G. On the origin of nonexponential fluorescence decay in tryptophan and its derivatives. J. Am. Chem. Soc. 1983, 105 (12), 3824 10.1021/ja00350a014.

[ref59] Swenberg C. E.; Geacintov N. E.; Breton J. Laser Pulse Excitation Studies of the Fluorescence of Chloroplasts. Photochem. Photobiol. 1978, 28 (6), 999 10.1111/j.1751-1097.1978.tb07738.x.

[ref60] Fasman G. D. CRC Handbook of Biochemistry and Molecular Biology: Proteins; CRC Press, 2018.

[ref61] Strain H. H.; Thomas M. R.; Katz J. J. Spectral absorption properties of ordinary and fully deuteriated chlorophylls a and b. Biochim. Biophys. Acta 1963, 75, 306 10.1016/0006-3002(63)90617-6. 14104939

[ref62] Gigant B.; Wang C.; Ravelli R. B. G.; Roussi F.; Steinmetz M. O.; Curmi P. A.; Sobel A.; Knossow M. Structural basis for the regulation of tubulin by vinblastine. Nature 2005, 435 (7041), 519 10.1038/nature03566. 15917812

[ref63] Hamblin M. R.; Huang Y.-Y.; Heiskanen V. Non-mammalian Hosts and Photobiomodulation: Do All Life-forms Respond to Light?. Photochem. Photobiol. 2019, 95 (1), 126 10.1111/php.12951. 29882348 PMC6286699

[ref64] Brédas J.-L.; Sargent E. H.; Scholes G. D. Photovoltaic concepts inspired by coherence effects in photosynthetic systems. Nat. Mater. 2017, 16 (1), 35 10.1038/nmat4767. 27994245

[ref65] Wiesenhofer H.; Beljonne D.; Scholes G. D.; Hennebicq E.; Brédas J.-L.; Zojer E. Limitations of the Förster Description of Singlet Exciton Migration: The Illustrative Example of Energy Transfer to Ketonic Defects in Ladder-type Poly(para-phenylenes). Adv. Funct. Mater. 2005, 15 (1), 155 10.1002/adfm.200400108.

[ref66] Khan Y. R.; Dykstra T. E.; Scholes G. D. Exploring the Förster limit in a small FRET pair. Chem. Phys. Lett. 2008, 461 (4), 305 10.1016/j.cplett.2008.07.023.

[ref67] Kalra A. P.; Patel S. D.; Bhuiyan A. F.; Preto J.; Scheuer K. G.; Mohammed U.; Lewis J. D.; Rezania V.; Shankar K.; Tuszynski J. A. Investigation of the Electrical Properties of Microtubule Ensembles under Cell-Like Conditions. Nanomaterials 2020, 10 (2), 265 10.3390/nano10020265. 32033331 PMC7075204

[ref68] Arnal I.; Wade R. H. How Does Taxol Stabilize Microtubules?. Current Biology 1995, 5 (8), 900 10.1016/S0960-9822(95)00180-1. 7583148

[ref69] Schiff P. B.; Fant J.; Horwitz S. B. Promotion of Microtubule Assembly *in Vitro* by Taxol. Nature 1979, 277 (5698), 665 10.1038/277665a0. 423966

[ref70] Amos L. A.; Löwe J. How Taxol® stabilises microtubule structure. Chemistry & Biology 1999, 6 (3), R65 10.1016/S1074-5521(99)89002-4. 10074470

