# Supplementary material for: Electronic Energy Migration in Microtubules
Source: ACS Cent Sci. 2023 Jan 12;9(3):352–61. doi: 10.1021/acscentsci.2c01114 (PMC10037452; doi:10.1021/acscentsci.2c01114)
Supplement: Supplementary file 1 — oc2c01114_si_001.pdf [file oc2c01114_si_001.pdf]

Supporting Information for
Electronic Energy Migration in Microtubules

Aarat P. Kalra [a], Alfy Benny [a], Sophie M. Travis [b], Eric A. Zizzi [c], Austin Morales-Sanchez [a], Daniel G. Oblinsky [a], Travis J. A. Craddock [d], Stuart R. Hameroff [e], M. Bruce MacIver [f], Jack A. Tuszyński [c, g, h], Sabine Petry [b], Roger Penrose [i], Gregory D. Scholes [a, *]
[a] Department of Chemistry, New Frick Chemistry Building, Princeton University, NJ 08544, USA
[b] Department of Molecular Biology, Schultz Laboratory, Princeton University, NJ 08544, USA
[c] Department of Mechanical and Aerospace Engineering (DIMEAS), Torino 10129, Italy
[d] Departments of Psychology & Neuroscience, Computer Science, and Clinical Immunology, Nova Southeastern University, Ft. Lauderdale, FL 33314, USA
[e] Department of Anesthesiology, Center for Consciousness Studies, University of Arizona, Tucson, Arizona, USA
[f] Department of Anesthesiology, Stanford University School of Medicine, Stanford, CA 94305, USA
[g] Department of Physics, University of Alberta, Edmonton, Alberta T6G 2E1, Canada
[h] Department of Oncology, University of Alberta, Edmonton, Alberta T6G 1Z2, Canada
[i] Mathematical Institute, Andrew Wiles Building, University of Oxford, Radcliffe Observatory Quarter, Woodstock Road, Oxford, OX2 6GG, United Kingdom
* Correspondence: gscholes@princeton.edu

Supporting Information Text

DOCKING OF ETOMIDATE, ISOFLURANE AND PICROTOXIN TO HUMAN TUBULIN

Homology Modelling. We built homology models of the human tubulin dimer starting from the corresponding amino acid sequences of the alpha-1A and beta-1 chains obtained from UniProt (accession codes Q71U36 and P07437 respectively) [1]. The central dimer of the pdb structure 3J6F corresponding to a sheet of a GDP-bound dynamic microtubule with a resolution of 4.90 Å was used as a template [2]. Homology models were build using the Homology Modelling tool of the MOE software [3], and the best model was chosen based on the GB/VI score [4]. Highly fluctuating C-terminal tails were excluded from the obtained model for all subsequent analyzes. The quality of the generated model was assessed by comparing the phi-psi plot and the QMeanDisCo score between the model and the 3J6F template [5].

Structure Equilibration. The obtained homology model was completed by adding the Mg2+ ion and the GTP and GDP ligands in their experimental binding positions as found in the template, after adjusting their protonation state using the Protonate3D tool in MOE at 300K and pH=7.4. The tubulin dimer was parametrized using the AMBER99SB-ILDN force field [6], while ligands were parametrized using GAFF [7] and their partial charges calculated using the AM1-BCC method [8]. Energy minimization, equilibration and production MD runs were carried out using GROMACS 2021.4 [9]. More in detail, the final model of the human tubulin dimer was energy minimized for 1000 steps, and subsequently equilibrated for 100 ps in the NVT ensemble using the v-rescale thermostat with the C-alpha atoms position-restrained with a force of 1000 kcal/mol/nm [10]. We then carried out a further 500-ps equilibration in the NPT ensemble using the v-rescale thermostat and the Berendsen barostat with the same position restraints as before [11]. We finally lifted the restraints and performed a production molecular dynamics run of 200 ns for further extended structural equilibration in the NTP ensemble using the v-rescale thermostat and Parrinello-Rahman barostat [12]. We regarded the first 100 ns of this production MD simulation as additional extended structure equilibration, and we extracted the dominant conformation states of the dimer as the centroids after clustering the last 100 ns of this MD production run. The cluster tool provided by GROMACS was used, with the cutoff value set to 0.15 nm. The subsequent docking simulations were run using these centroids as the target structure.

Site identification and docking. After determining the dominant conformations of the dimer from the MD ensemble, we performed a scan for potential binding sites on the dimer using the SiteFinder tool provided in MOE. To focus the analysis not only on the most likely binding sites, but also on pockets with a potentially lower affinity, we investigated all predicted binding sites with a positive PLB score [13], and docked the four different ligands within these regions. In addition, to further complement the analysis, the ligands were also docked to three experimentally known and clinically relevant binding sites on the tubulin dimer, namely the (a) Colchicine binding site, located near the inter-monomer interface and defined by loop βT7, helix βH8, strands βS8 and βS9 and the αT5 loop; (b) the taxane binding site, located on the β subunit surrounded by helix H1, the H6-H7 loop, the H7 helix, the M loop and the S9-S10 loop; this site is partially overlapping with site number 5 found by the SiteFinder tool (c) the vinca alkaloid binding site, at the longitudinal dimer-dimer interface within protofilaments, surrounded by loop αT7, helix αH10 and strand αS9.

Docking runs were carried out in MOE, with the following methodology: we performed the initial placement of the ligands using the triangle matcher algorithm, which describes the active site using α spheres. The generated poses were then scored using the London dG scoring function [3]. Subsequently, for each ligand, the 30 best poses found using the triangle matcher algorithm were further energy-minimized starting from their initial placement, using a Molecular Mechanics approach with the protein kept rigid. The final poses were finally re-scored using the GBVI/WSA dG scoring function [14], and the top 5 poses were considered for analysis.

Results

(A) Quality of the Homology Models. The human alpha 1A and beta 1 tubulin chains shared a sequence identity of 99.3% and 97.9% with the corresponding chains of the 3J6F template, respectively, thus ensuring the reliability of the homology modelling approach. Indeed, on the phi-psi plot, the generated human alpha/beta dimer model featured 94.18% of residues in core regions, 5.35% in allowed regions and 0.47% outliers. In comparison, the same figures for the 3J6F template were 96.81%, 2.83% and 0.36% respectively. The QMeanDisCo global score of the model was 0.76 ± 0.05, which is comparable within error to the corresponding score of the crystallographic template (0.79 ± 0.05) (see Figure S12: comparison of QMean scores). Both quality assessments confirm the comparable structural quality of the homology model with respect to its 3J6F template.

(B) Docking. Clustering of the last 100 ns of the MD ensemble at a 0.15 nm RMSD cutoff yielded a single cluster, suggesting overall conformational stability and no major rearrangements in the protein structure. The centroid of this cluster, representing the dominant conformational state of the tubulin dimer throughout the simulation, was chosen as the conformation to perform docking against.
First, we scanned for possible binding sites on the tubulin dimer using the SiteFinder tool in MOE, and found a total of 43 putative binding sites, with PLB scores ranging from 3.66 (best) to -0.79 (worst). All binding sites with positive PLB values were kept as docking sites, resulting in a total of 13 analyzed binding sites. A summary of these binding sites is provided in Table S6 together with the experimentally known binding sites on tubulin for colchicine, taxanes and vinca alkaloids. In the following, the binding site numbering of Table S6 will be used to identify the sites.

Etomidate Predicted binding energies for Etomidate range from -6.77 to -4.36 kcal/mol (mean±std:-5.71±0.51 kcal/mol). The best binding energy was obtained on site 5 (-6.77 kcal/mol), and the interaction between Etomidate and the protein in this site is highlighted in Figure S9A below. The average results in all 16 binding sites are summarized in Table S7.

Isoflurane Predicted binding energies for Isoflurane range from -4.64 to -3.66 kcal/mol (mean±std:-4.11±0.22 kcal/mol), indicative of a slightly weaker predicted interaction with respect to etomidate. The best binding energy was obtained on site 3 (-4.64 kcal/mol), and the corresponding interaction between isoflurane and the protein in this site is highlighted in Figure S9b, although it is to be underlined how the differences between the different binding poses and binding sites are realistically all within the error of the docking methodology. The average results in all 16 binding sites are summarized in Table S8.

Picrotoxinin Predicted binding energies for Picrotoxinin range from -5.88 to 0.56 kcal/mol (mean±std: 4.22±1.79 kcal/mol). The best binding energy was obtained on the taxol binding site (-5.88/ kcal/mol), and the corresponding interaction between the ligand and the protein in this site is highlighted in Figure S9C. The average results in all 16 binding sites are summarized in Table S9.

Picrotin Predicted binding energies for Picrotin range from -5.72 to -2.82 kcal/mol (mean±std:-4.89±0.64 kacl/mol). The best binding energy was obtained in the taxol binding site (-5.72 kcal/mol), and the corresponding interaction between the ligand and the protein in this site is highlighted in Figure S9D. The average results in all 16 binding sites are summarized in Table S10.

Summary of docking studies. Overall, for all compounds except isoflurane, the highest predicted affinity is consistently found to be in or near (site 5) the taxol binding site, which is a known and relevant binding site on the tubulin dimer. Predicted energies in the other putative binding locations are nevertheless similar and most are within the thermal noise level of 0.6 kcal/mol. Hence, no hard conclusions can be drawn regarding a distinctive preferred binding site for either of the analyzed ligands on human tubulin.

Picrotoxin is a 1:1 molar mixture of picrotin and picrotoxinin, two highly similar compounds. Hence, docking was performed for both ligands, and no substantial difference in predicted binding affinities emerged, coherently with their high chemical similarity. No conclusions can be drawn from the docking simulations to distinguish a more active compound on tubulin among the two; in fact, both compounds showed the best predicted dG value in the taxol binding site, where they are predicted to form at least two hydrogen bonds with residues ARG318, ARG359 and ARG276 with differences well within the error of docking predictions.

KINETIC MONTE CARLO SIMULATIONS

Preparation of microtubule crystal structure We constructed the all-atom structure of the full microtubule using the following protocol. Firstly, the tubulin sheet of PDB structure 7SJ7 [15] was preprocessed using MOE [3] to fix missing residues in the structure, assign the correct protonation states and perform energy minimization to relief atomic clashes. We then extracted the central tubulin dimer of this structure and used it to construct an initial template of a single tubulin ring, composed of 13 dimers, by fitting 13 copies of the dimer onto the original electron density map deposited in the Electron Microscopy Data Bank (accession code EMD-25156), using the "*Fit in Map*" tool of UCSF ChimeraX [16].
To construct the final microtubule, we first carried out 200-ns Molecular Dynamics simulations of the tubulin sheet to equilibrate the structure and obtain a diversified structural ensemble. In detail, simulations were carried out in GROMACS 2021.4 [9] in the NPT ensemble, using the AMBER 99SB-ILDN force field [6], PME electrostatics with a cutoff of 1.2 nm, the velocity rescale thermostat [10] at 300K and the Parrinello-Rahman barostat [12]. We subsequently clustered the last 150 ns of this MD simulation with a cutoff of 0.10 nm using the *gmx cluster* tool, and extracted the centroids of each cluster, focusing only on the central dimer of the tubulin sheet. These centroids represent a set of dominant conformations of the tubulin dimer within a microtubule, and were used to build the final microtubule. In greater detail, we generated each of the final tubulin rings by randomly choosing centroids from the MD clusters and RMSD-fitting them onto the template tubulin ring described above. This was repeated for each of the 31 tubulin rings composing the final microtubule, which was assembled by spacing the rings axially by 8.15 nm, as described in earlier literature for the 13:3B MT lattice [17].
In summary, we eventually obtained a final B-lattice microtubule with a length of 252.65 nm and composed of an ensemble of different tubulin dimer conformations, extracted from all-atom MD simulations and representative of the conformational heterogeneity of tubulin within a microtubule.

Simulation protocol for homo- and hetero-transfer among tryptophan and tyrosine residues. The monomers of TYR and TRP were optimized in the CAM-B3LYP/AUG-CC-PVDZ level of theory and the optimized geometry was used to calculate the transition dipole moments using TDDFT (CAM-B3LYP/AUG-CC-PVDZ). The calculations were carried out in Gaussian 16 program package [18].The obtained transition dipole moments were reoriented at the respective molecular coordinates for calculating the long-range coulombic coupling strengths in the 31-long dimer microtubule crystal structure. We took the dissipation rate (k_{Dis}) as 3.06×10^8 s^{-1} [19].

Charge transfer Integral calculations were carried out on 5 representative TYR dimers selected from the microtubule crystal structure with Inter-aromatic (R_{IA}) distances below 6.3 Å. A constrained optimization (B3LYP/6-311+G(d)) were performed on the selected TYR dimers by freezing the carbon skeleton prior to charge transfer integral calculations. The charge transfers Integral calculations (B3LYP/6-311+G(d)) were carried out using the dimer projection (DIPRO) method [20] using the CATNIP program package [21] available from https://github.com/JoshuaSBrown/QC_Tools).

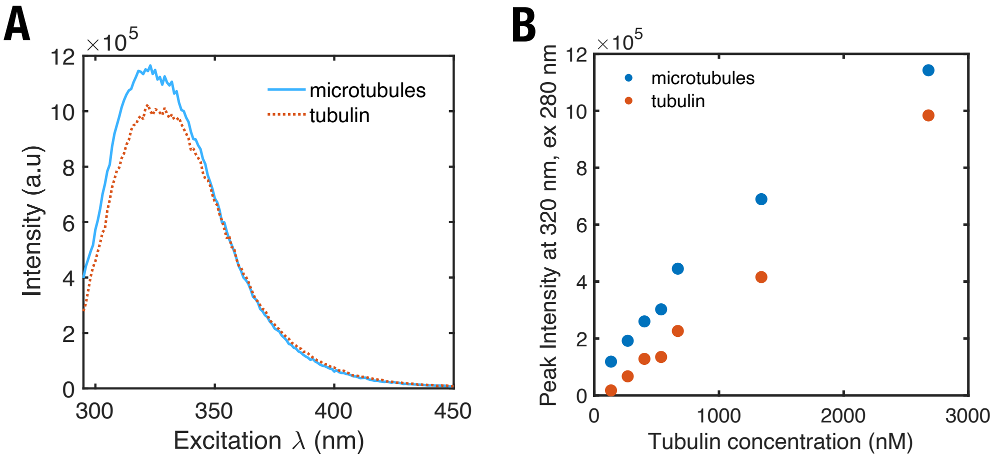

Figure S1. Emission profiles of tubulin and microtubules. (A), The emission spectrum of tubulin and microtubules as a function of concentration, (B), Variation of peak fluorescence intensity at different tubulin concentrations. All samples were excited using λexcitation = 280 nm.

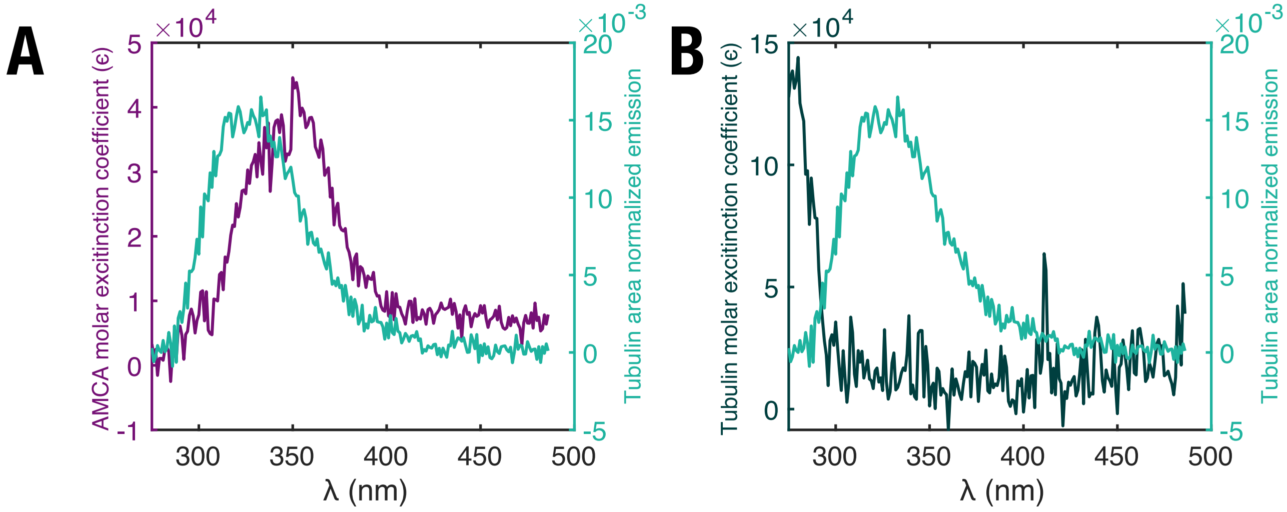

Figure S2. Molar extinction coefficient and area normalized emission spectra of (A) tubulin tryptophan and AMCA, (B) tubulin tryptophan for inter-tryptophan energy transfer.

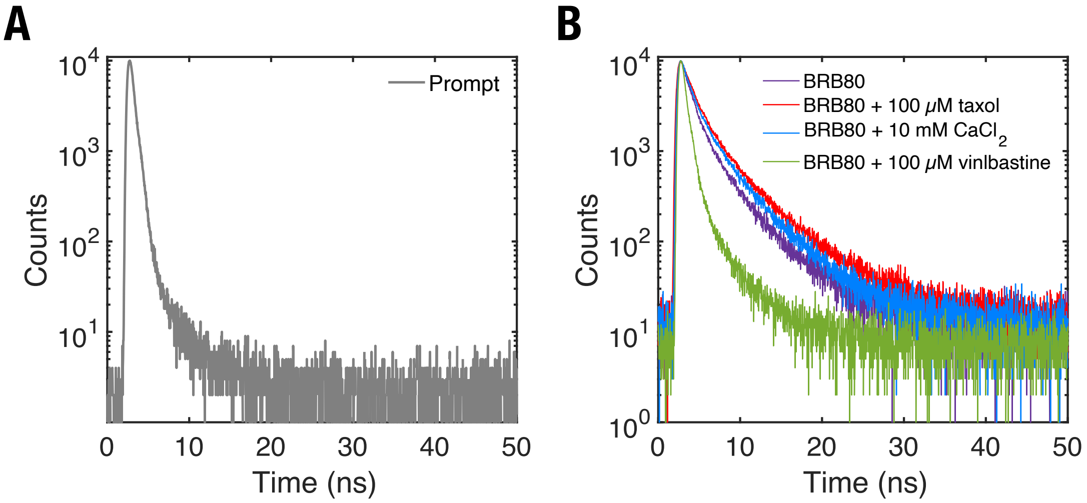

Figure S3. (A), Representative Instrument Response Function (IRF; 'prompt') signal extracted from a ludox colloidal solution, (B), Representative signal from a BRB80, BRB80T (background for microtubule containing solutions), BRB80Ca (background for free GTP-tubulin containing solutions), and BRB80V (background for GTP-tubulin oligomer containing solutions). BRB80V had characteristic lifetimes of 3.67 ns (10.14%), 0.85 ns (5.22%) and 53.6 ps (84.64%).

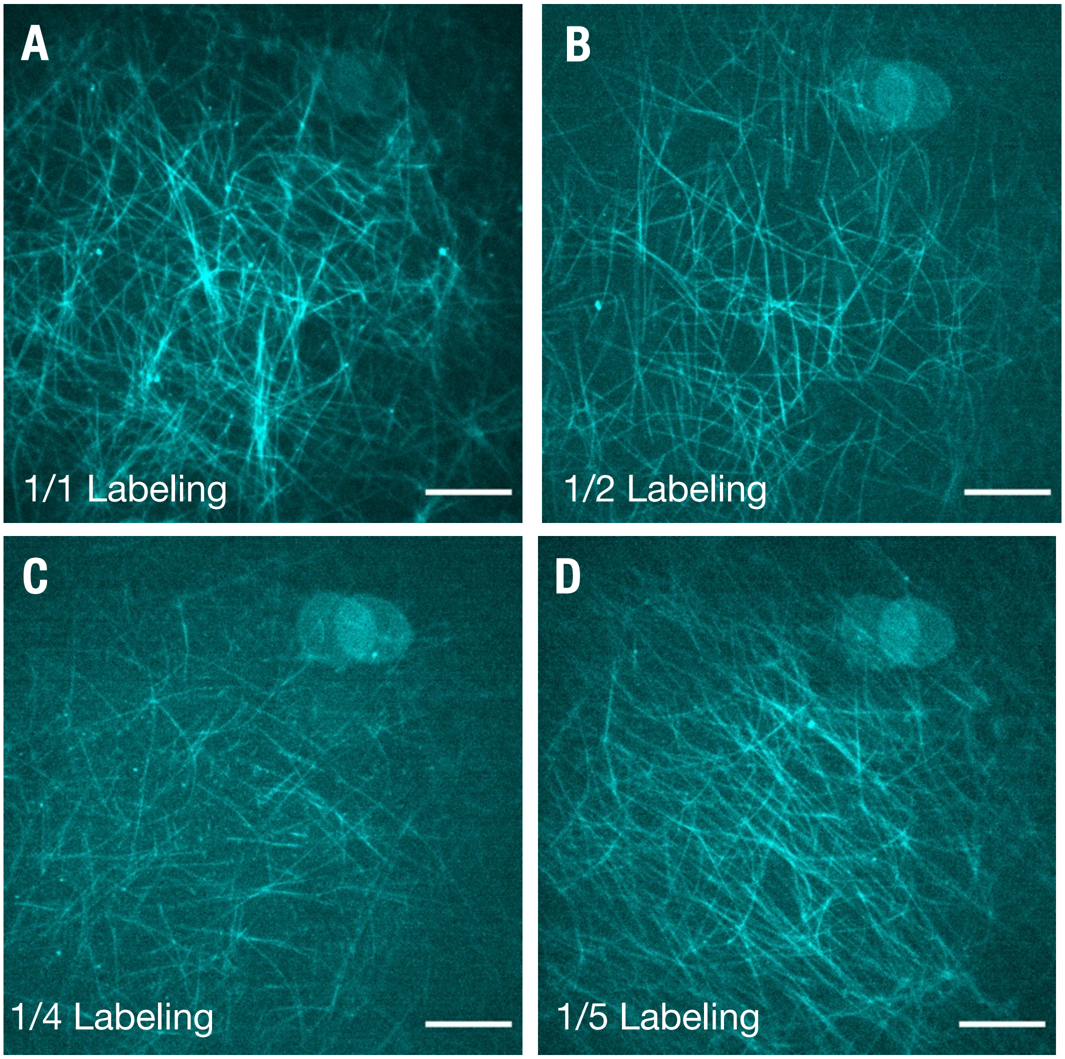

Figure S4. Experimentally evaluating the polymerization of microtubules with differing AMCA concentrations using confocal fluorescence microscopy. Microtubules polymerized using (A), 1/1 labeling ratio (AMCA concentration 2.27 μM in solution; tubulin concentration 2.27 μM in solution), (B), 1/2 labeling ratio (AMCA concentration 1.135 μM), (C), 1/4 labeling ratio (AMCA concentration 0.57 μM) and (D), 1/5 labeling ratio (AMCA concentration 0.45 μM). Scale bars represent 10 μm. While a limited amount of microtubule bundling can be seen in (A) and (D), no bundling was observed in the larger solution.

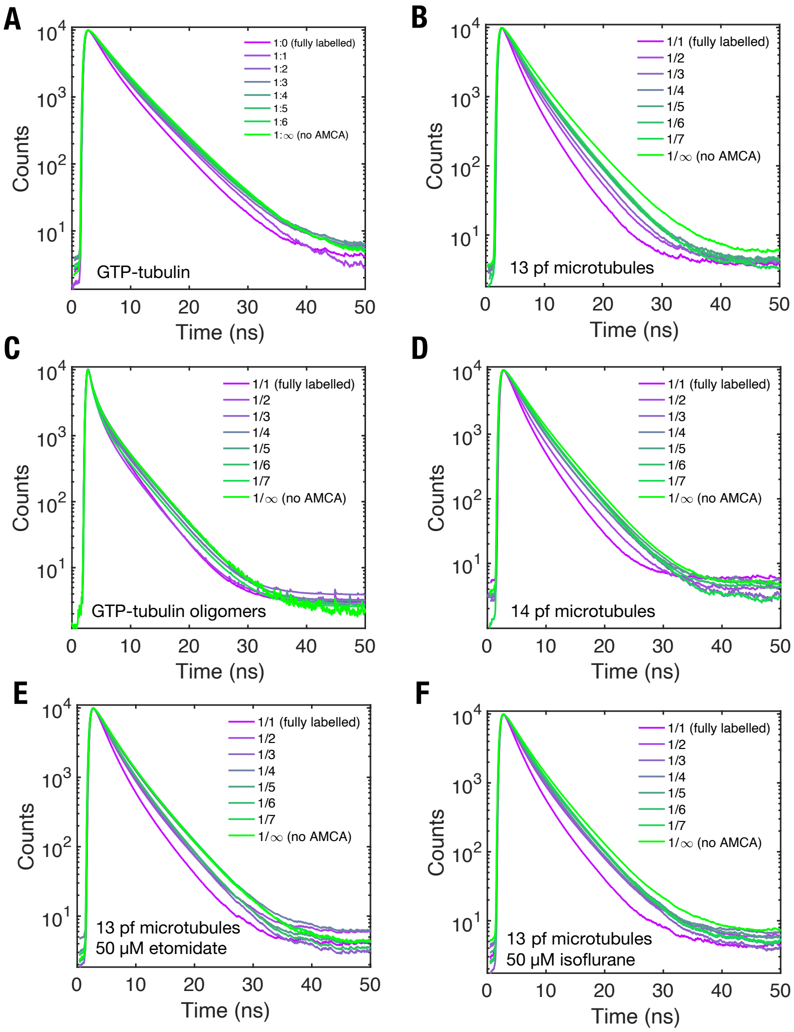

Figure S5. Time resolved fluorescence spectroscopy showing the influence of AMCA labeling ratio on tryptophan fluorescence lifetimes in (A), free GTP-tubulin in solution, (B), 13 protofilament microtubules, (C), GTP-tubulin oligomers, (D), 14 protofilament microtubules (E), 13 protofilament microtubules in the presence of 50 μM etomidate, (F), free 13 protofilament microtubules in the presence of 50 μM isoflurane, as a function of AMCA labeling ratio.

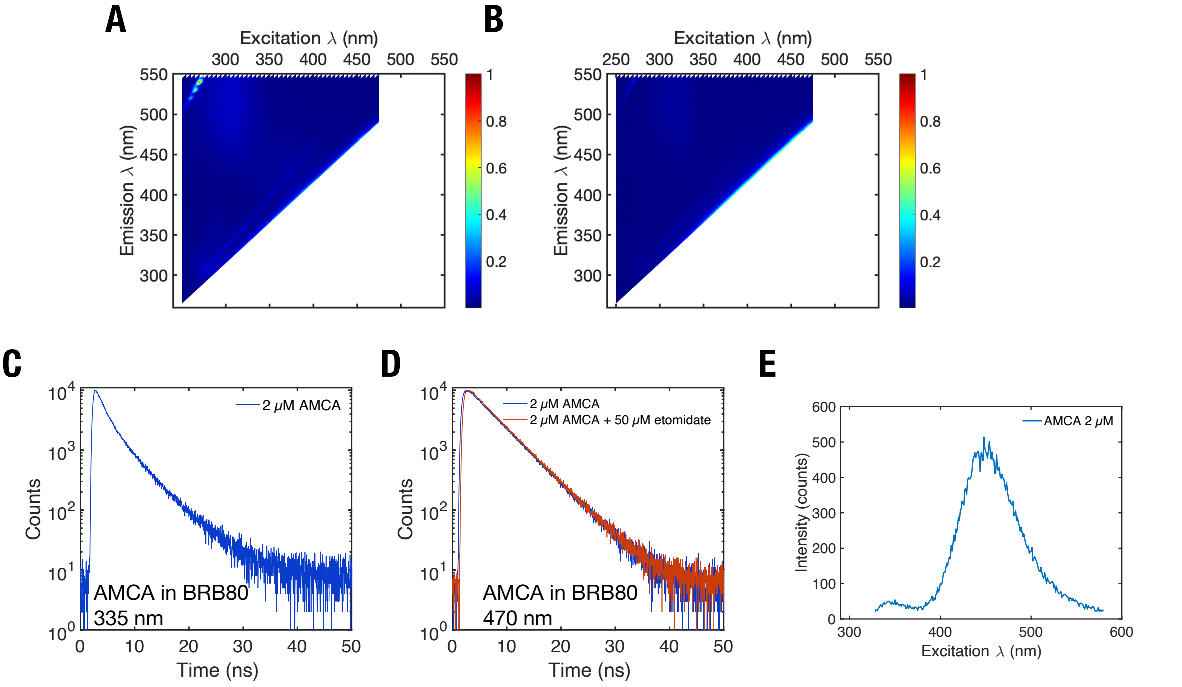

Figure S6. Intensity normalized fluorescence spectra of (A), 50 μM etomidate in BRB80 showing highest fluorescence emission at Raman scattering peaks of water, (B), 50 μM isoflurane in BRB80 showing highest fluorescence emission at Raman scattering peaks of water. These spectra show that etomidate and isoflurane contribute to negligible fluorescence in the excitation 270-310 nm and emission 300-350 nm range, where tryptophan is active. (C), Representative signal from a 2 μM AMCA solution, measured at excitation 305 nm, emission 335 nm (conditions and background solution condition identical to that of GTP-tubulin polymerized microtubules), (D), 470 nm in the presence of agents etomidate, isoflurane and picrotoxin, (E), Steady-state emission spectrum of AMCA at excitation 305 nm recorded using the TCSPC instrumentation.

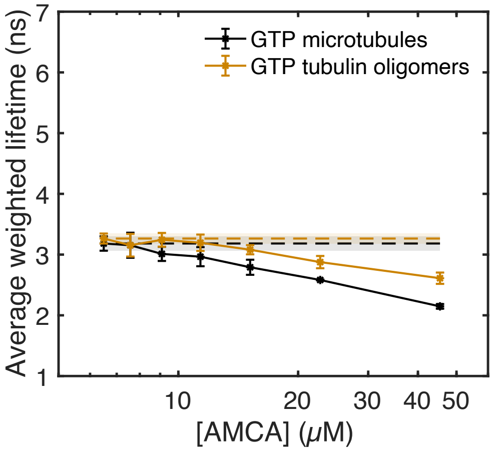

Figure S7. Average weighted tryptophan fluorescence lifetime of GTP tubulin oligomers as a function of AMCA concentration.

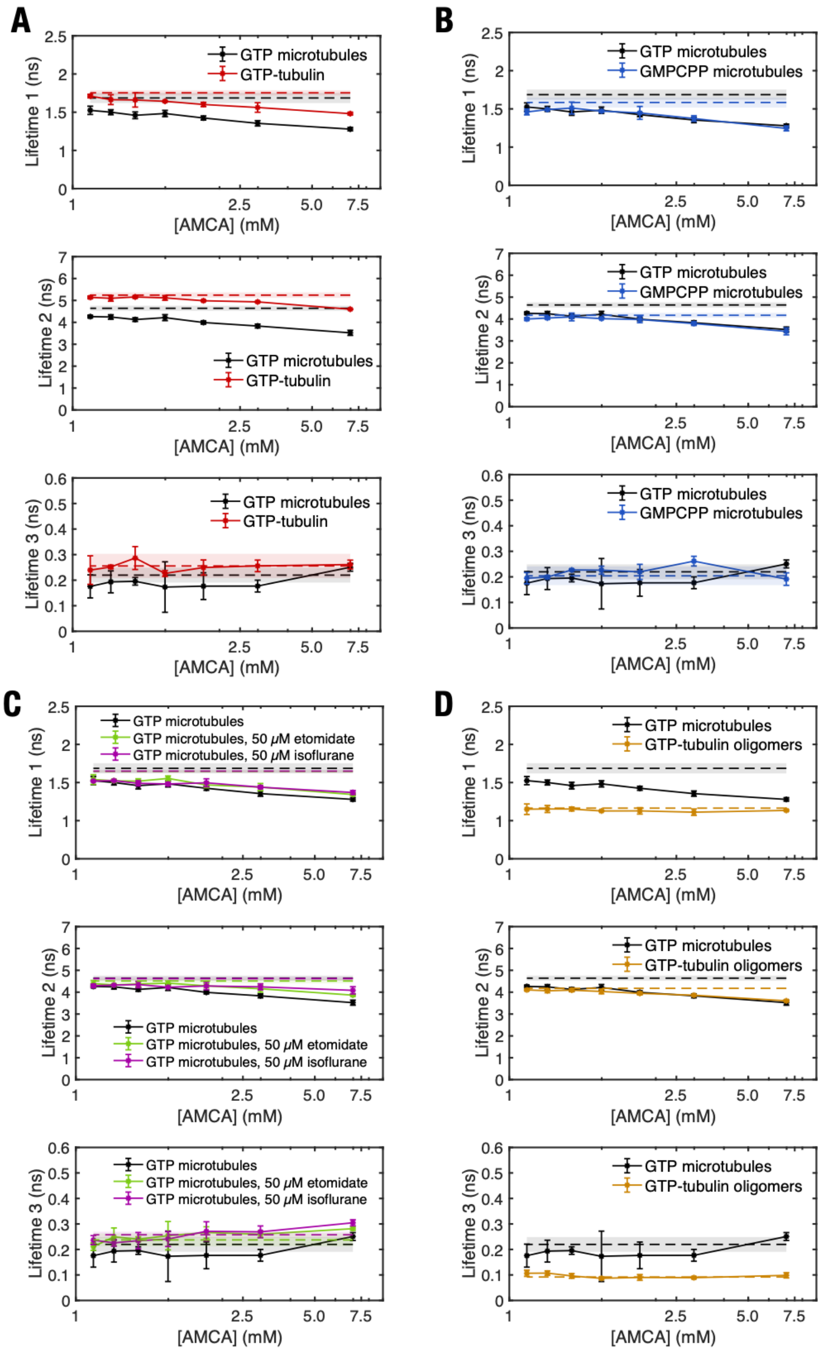

Figure S8. Tryptophan fluorescence lifetimes of different tubulin polymers as a function of AMCA concentration Fitted lifetimes of tryptophan fluorescence as a function of AMCA labeling ratio for (A), GTP-tubulin compared to that of 13 protofilament microtubules, (B), 14 protofilament microtubules compared to that of 13 protofilament microtubules, (C), GTP-tubulin oligomers compared to that of 13 protofilament microtubules, (D), 13 protofilament microtubules in the presence of etomidate and isoflurane compared to that of 13 protofilament microtubules in their absence.

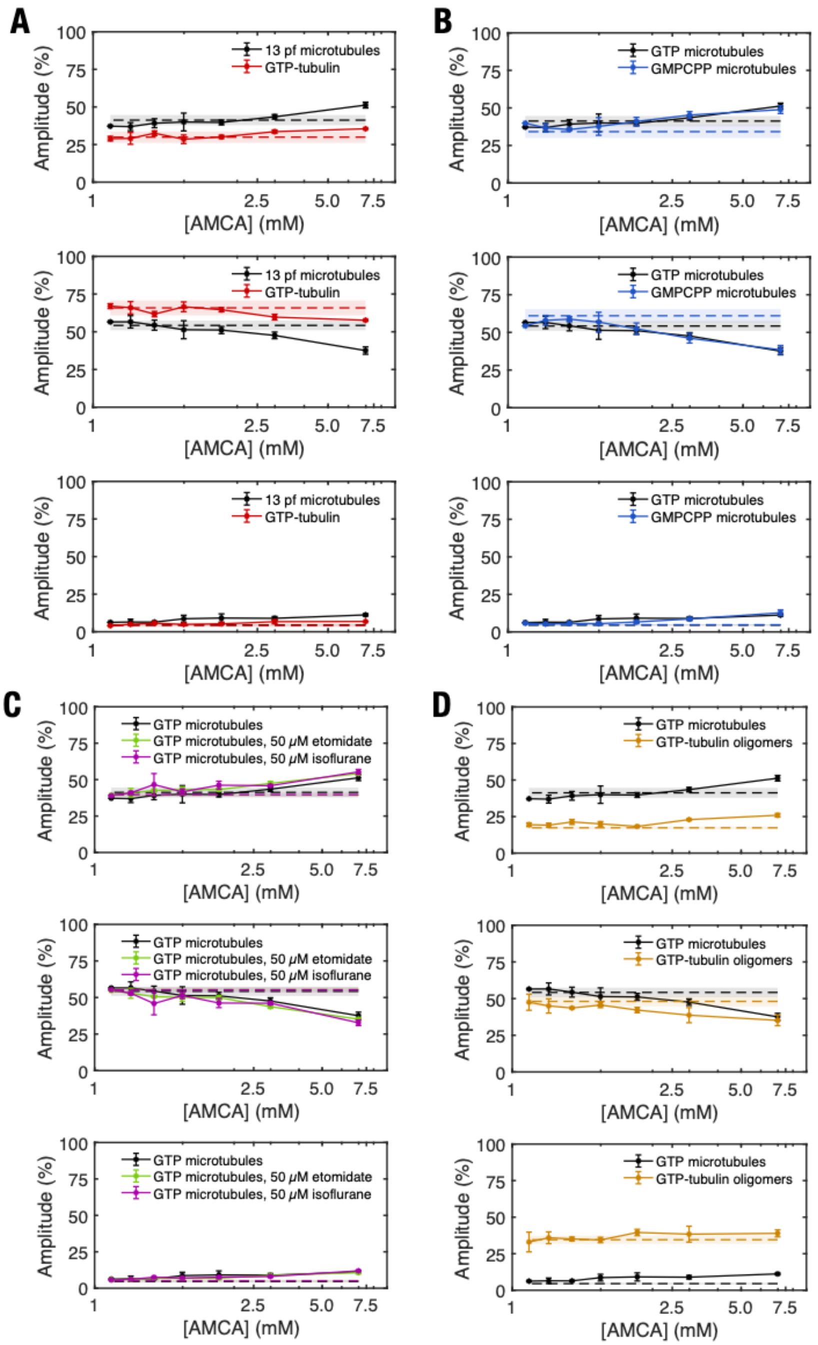

Figure S9. Relative contributions of each lifetime component in tryptophan fluorescence for different tubulin polymers as a function of AMCA concentration. Fitted amplitudes of tryptophan fluorescence as a function of AMCA labeling ratio for (A), GTP-tubulin compared to that of 13 protofilament microtubules, (B), 14 protofilament microtubules compared to that of 13 protofilament microtubules, (C), GTP-tubulin oligomers compared to that of 13 protofilament microtubules, (D), 13 protofilament microtubules in the presence of etomidate and isoflurane compared to that of 13 protofilament microtubules in their absence.

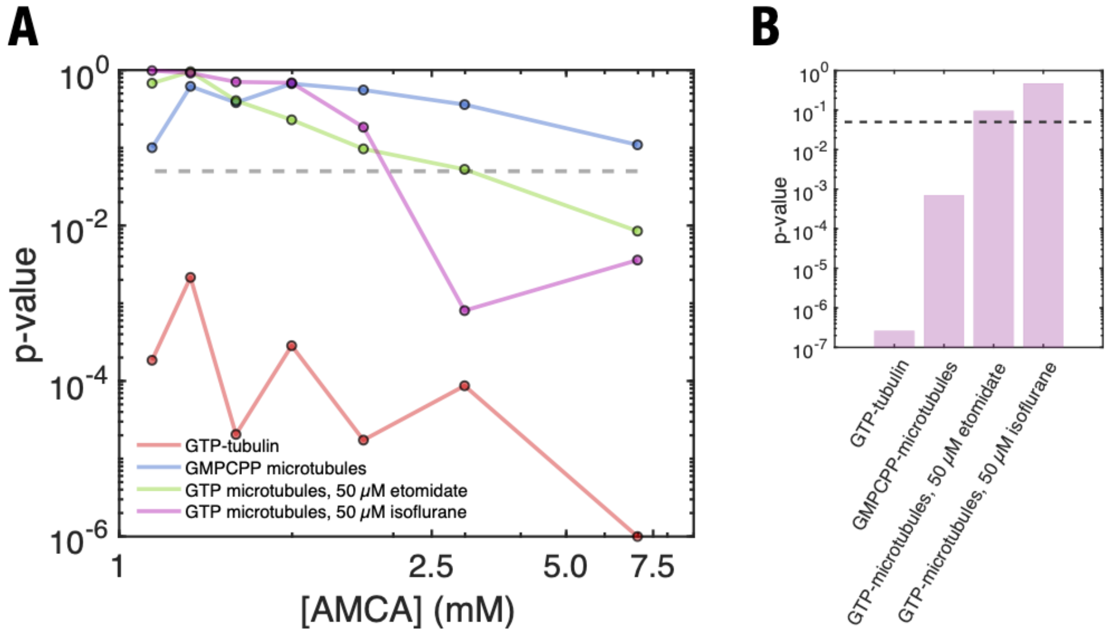

Figure S10. (A) p-values obtained from a two tailed t-test, comparing the values of average weighted lifetimes obtained for GTP-microtubules, to those of GTP-tubulin, GMPCPP microtubules, GTP microtubules in the presence of 50 μM etomidate and GTP microtubules in the presence of 50 μM isoflurane in the presence of varying concentrations of AMCA. The dashed grey line represents a p-value of 0.05. (B) p-values obtained by comparing the average weighted lifetimes obtained for unlabeled microtubules with those of unlabeled GTP-tubulin, unlabeled GMPCPP microtubules, unlabeled GTP microtubules in the presence of 50 μM etomidate and unlabeled GTP microtubules in the presence of 50 μM isoflurane. The dashed line represents a p-value of 0.05.

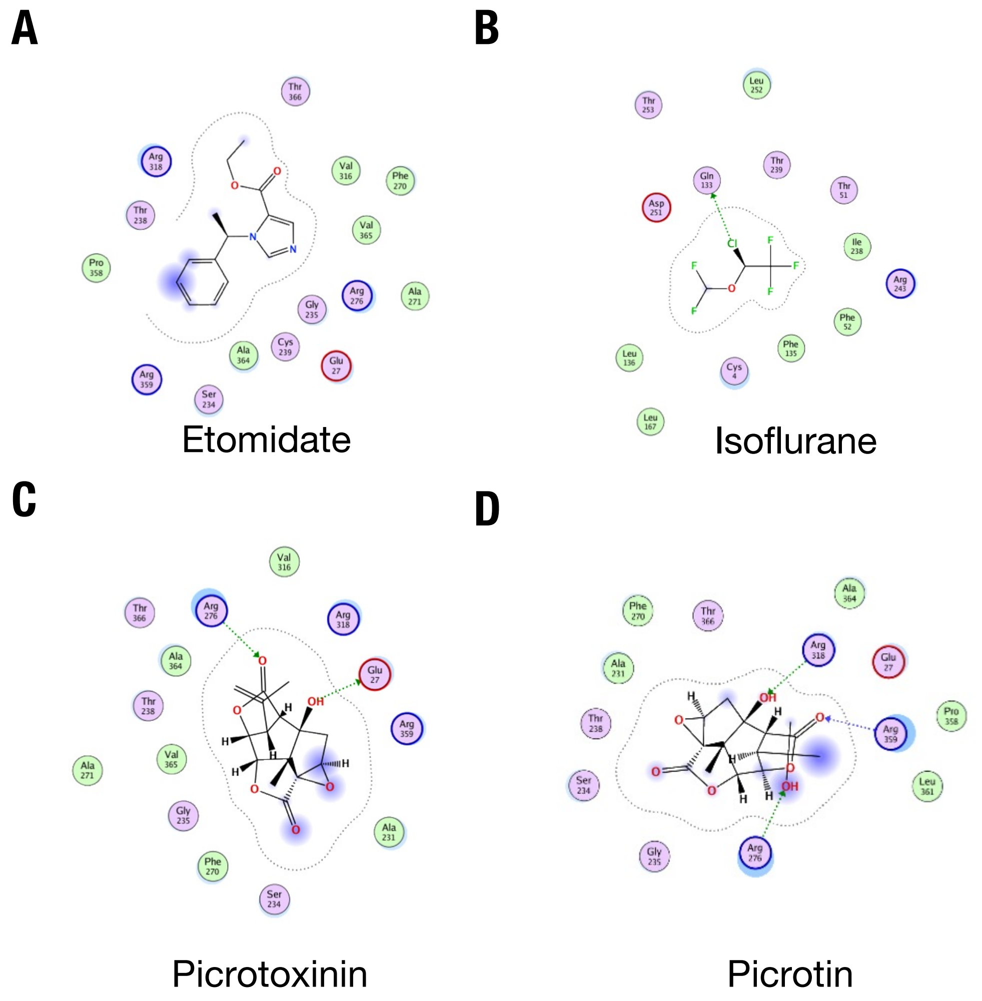

Figure S11. (A), Weak interactions between etomidate and binding site residues of Site 5, which featured the best predicted binding energy, (B), Detailed interactions between isoflurane and binding site residues of Site 3, which featured the best predicted binding energy, (C), Interactions between picrotoxinin and the taxol binding site, which featured the best predicted binding energy, d, Interactions between picrotin and binding site residues of the taxol binding site, which featured the best predicted binding energy.

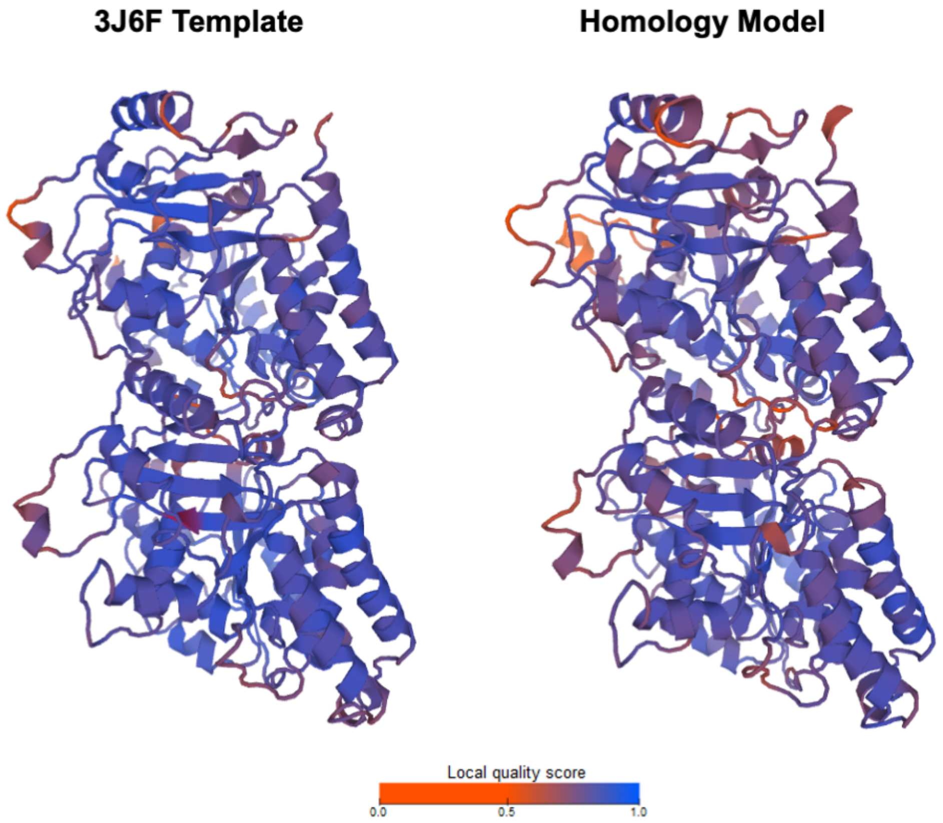

Figure S12. Visual comparison of the distribution of QMeanDisCo scores of the template (PDB ID 3J6F, left) and the final homology model (right).

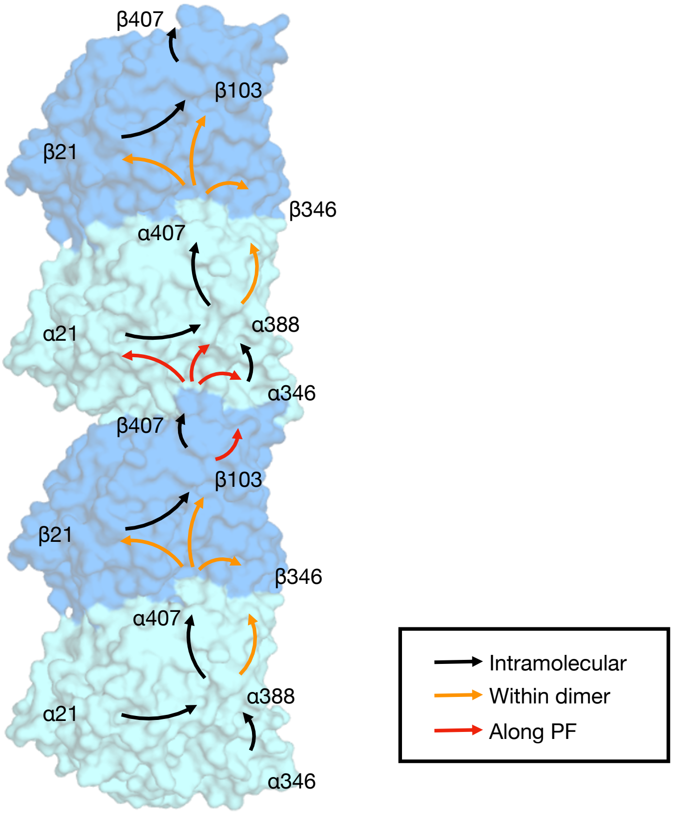

Figure S13. Schematic showing long-range energy transport along a microtubule.

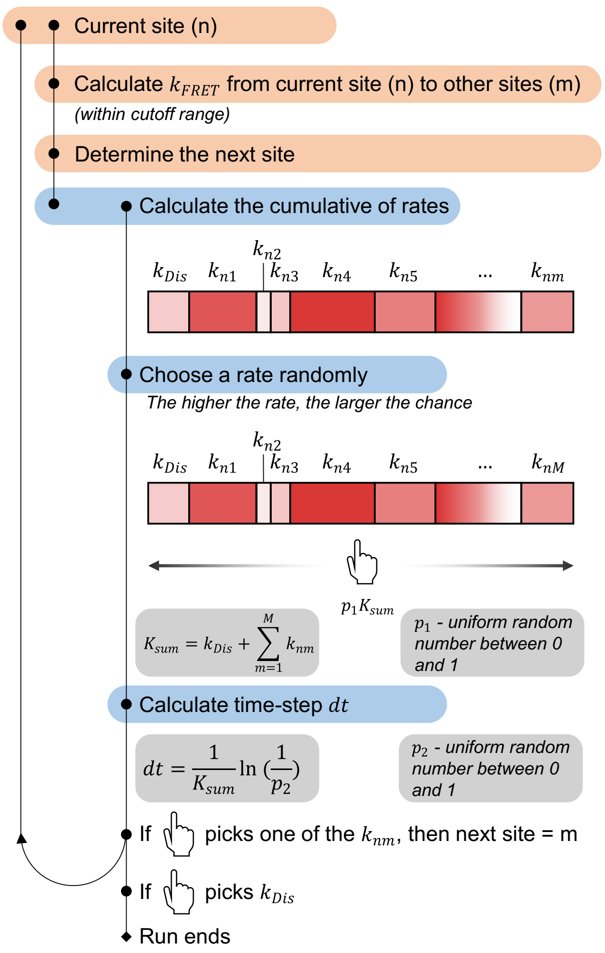

Figure S14. Algorithm used for kinetic Monte Carlo simulations.

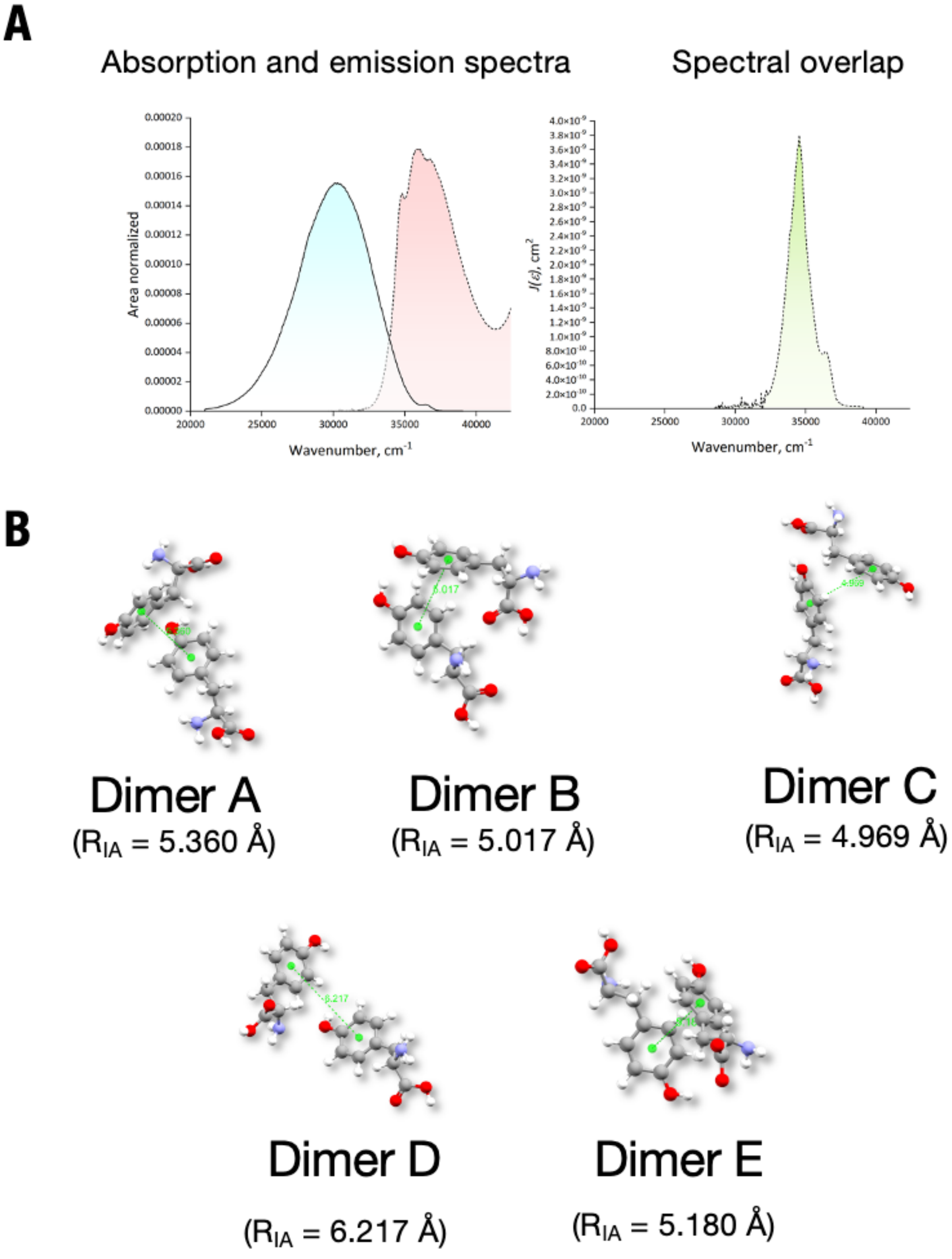

Figure S15. (A) Spectral overlap integral values for photoexcitation donor acceptor pairs (B) Graphs showing absorption and emission spectra used for calculations. (C) Representative TYR dimers with short inter-aromatic distance (R_{IA}) selected for calculating hole and electron transfer integrals.

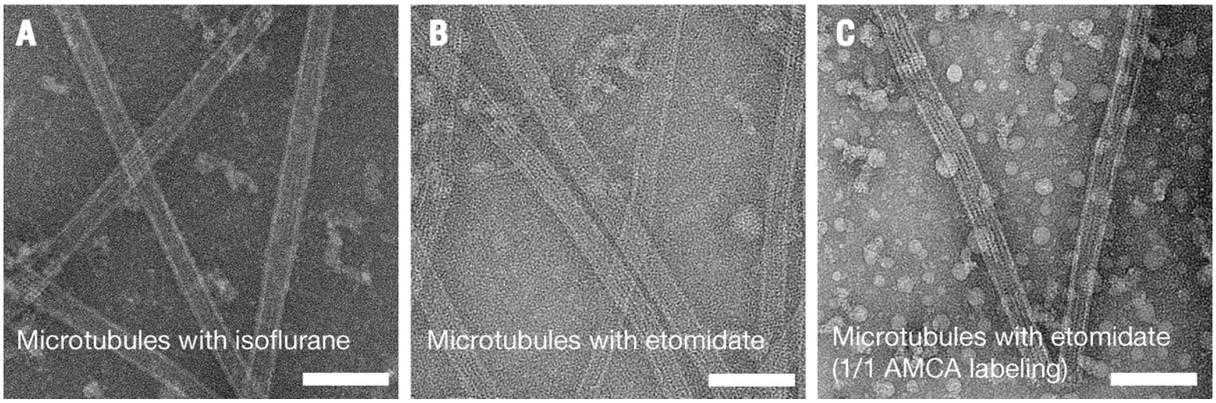

Figure S16. TEM validating microtubule presence in anesthetic containing solutions. Paclitaxel stabilized microtubules in the presence of (*A*) 50 µM isoflurane, (*B*) 50 µM etomidate (*C*) 50 µM etomidate and microtubules polymerized using tubulin labeled with AMCA. Scale bars represent 100 nm.

Table S1. Nearest neighbor distances (Å) between tryptophan residues in different tubulin polymers. CD2 (carbon at the delta-2 position) atoms in pairs of tryptophan residues were used to measure distances between residues.

	**14 protofilament microtubules (6DPU)**	**GTP-tubulin (3J6G)**	**13 protofilament microtubules (3J6G)**	**GTP-tubulin oligomers (Vinblastine stabilized)**
TUBα1 W21	27.9	28	27.7	26.6
TUBα1 W346	14.3	19.9	15.2	13.8
TUBα1 W388	17.9	19.9	19.9	20.3
TUBα1 W407	15.8	15.9	15.9	15.9
TUBβ W21	26.8	26.3	26.3	26.2
TUBβ W103	13.9	14.3	14.3	13.5
TUBβ W346	15.8	15.9	15.9	15.9
TUBβ W407	13.9	14.3	14.3	13.5
**average min**	**18.2875**	**19.3125**	**18.6875**	**18.2125**
**std min**	**5.7543617**	**5.325528143**	**5.435974745**	**5.51735897**

Table S2. Distances (Å) between tryptophan residues for 13 and 14 protofilament microtubules.

**Residue 1**	**Residue 2**	**14 protofilament microtubule (6DPU) CD2 Distance (Å)**	**13 protofilament microtubule (3J6G) CD2 Distance (Å)**
	**TUBα1 Intradimer**		
TUBα1 W21	TUBα1 W346	36.8	37.6
TUBα1 W21	TUBα1 W388	29.2	28
TUBα1 W21	TUBα1 W407	35.4	35
TUBα1 W346	TUBα1 W388	17.9	19.9
TUBα1 W346	TUBα1 W407	42.8	43.5
TUBα1 W388	TUBα1 W407	25.8	24.4
	**TUBβ Intradimer**		
Tubβ W21	Tubβ W103	26.8	26.3
Tubβ W21	Tubβ W346	38	37.9
Tubβ W21	Tubβ W407	35.3	35.2
Tubβ W103	Tubβ W346	33.8	33.5
Tubβ W103	Tubβ W407	13.9	14.3
Tubβ W346	Tubβ W407	45.7	45.5
	**TUBα1 to TUBβ across dimer**		
TUBα1 W21	Tubβ W21	41	40.8
TUBα1 W21	Tubβ W103	59.3	58.3
TUBα1 W21	Tubβ W346	43.3	43
TUBα1 W21	Tubβ W407	71.9	71.4
TUBα1 W346	Tubβ W21	65.9	65.7
TUBα1 W346	Tubβ W103	72.3	70.7
TUBα1 W346	Tubβ W346	41.7	40.8
TUBα1 W346	Tubβ W407	85.5	84.3
TUBα1 W388	Tubβ W21	49.2	46.5
TUBα1 W388	Tubβ W103	72.3	51.5
TUBα1 W388	Tubβ W346	25	23
TUBα1 W388	Tubβ W407	67.8	65.1
TUBα1 W407	Tubβ W21	27.7	27.7
TUBα1 W407	Tubβ W103	26.9	27.7
TUBα1 W407	Tubβ W346	15.8	15.9
TUBα1 W407	Tubβ W407	42	41.7
	**Tubα1 to TUBβ along PROTOFILAMENT**		
TUBα1 W21	Tubβ W21	42.9	42.7
TUBα1 W21	Tubβ W103	37.9	38
TUBα1 W21	Tubβ W346	68.1	67.5
TUBα1 W21	Tubβ W407	27.9	27.7
TUBα1 W346	Tubβ W21	68.1	43.9

TUBα1 W346	Tubβ W103	20.7	22
TUBα1 W346	Tubβ W346	42.3	42.7
TUBα1 W346	Tubβ W407	14.3	15.2
TUBα1 W388	Tubβ W21	52.8	53.9
TUBα1 W388	Tubβ W103	33.4	35.7
TUBα1 W388	Tubβ W346	60	61.8
TUBα1 W388	Tubβ W407	20.4	22.1
TUBα1 W407	Tubβ W21	72.9	72.4
TUBα1 W407	Tubβ W103	55.8	55.8
TUBα1 W407	Tubβ W346	85.6	85.3
TUBα1 W407	Tubβ W407	42	41.6
	**Tubα1 to TUBα1 across lattice**		
TUBα1 W21	TUBα1 W21	48.6	48.6
TUBα1 W21	TUBα1 W346	79.2	79.8
TUBα1 W21	TUBα1 W388	71.9	70.3
TUBα1 W21	TUBα1 W407	63.7	63.6
TUBα1 W346	TUBα1 W21	48.4	50.4
TUBα1 W346	TUBα1 W346	61.4	62.3
TUBα1 W346	TUBα1 W388	58.6	59.4
TUBα1 W346	TUBα1 W407	57.5	58.4
TUBα1 W388	TUBα1 W21	48.1	48.7
TUBα1 W388	TUBα1 W346	67.4	68.3
TUBα1 W388	TUBα1 W388	59.9	59.5
TUBα1 W388	TUBα1 W407	50.6	50.7
TUBα1 W407	TUBα1 W21	64.5	64.1
TUBα1 W407	TUBα1 W346	88.6	87.6
TUBα1 W407	TUBα1 W388	77.1	74.8
TUBα1 W407	TUBα1 W407	58.9	58.8
	**Tubβ to TUBβ across lattice**		
Tubβ W21	Tubβ W21	48.9	48.6
Tubβ W21	Tubβ W103	60.4	59.7
Tubβ W21	Tubβ W346	81.2	81.1
Tubβ W21	Tubβ W407	63.7	63.1
Tubβ W103	Tubβ W21	59.6	59.4
Tubβ W103	Tubβ W103	59	58.7
Tubβ W103	Tubβ W346	84.9	85
Tubβ W103	Tubβ W407	60.9	59.7
Tubβ W346	Tubβ W21	47.8	47.2

Tubβ W346	Tubβ W103	48.3	47
Tubβ W346	Tubβ W346	62	61.8
Tubβ W346	Tubβ W407	56.7	55.6
Tubβ W407	Tubβ W21	64.4	64.5
Tubβ W407	Tubβ W103	60.9	60.9
Tubβ W407	Tubβ W346	90.4	90.6
Tubβ W407	Tubβ W407	58.6	58.4

Table S4. Estimation of 2D diffusion length using Stern-Volmer analysis.

Variable	Symbol	Value	Estimation method
Stern-Volmer quenching constant	k_Q	See Table 1	TCSPC analysis
Tryptophan weighted average lifetime in tubulin polymer in the absence of AMCA	τ_0	See Figure 4	TCSPC
Tryptophan weighted average lifetime in tubulin polymer as a function of [AMCA]	τ	See Figure 4	TCSPC
sum of the radii of tryptophan and AMCA	r	1 nm	approximate sum

Table S5. Comparison of 2D diffusion lengths as estimated by Stern-Volmer kinetics and RET using Förster theory.

**Tubulin polymerization state**	**L_{SV} (nm)**
Free GTP-tubulin	4.07 ± 0.1
13 protofilament microtubules (GTP-tubulin polymerized)	6.64 ± 0.1
14 protofilament microtubules (GMPCPP-tubulin polymerized)	6.13 ± 0.1
GTP-tubulin oligomers	4.63 ± 0.1
13 protofilament microtubules with 50 μM etomidate	5.61 ± 0.1
13 protofilament microtubules with 50 μM isoflurane	5.81 ± 0.2

Table S6. Summary of all binding sites found by SiteFinder with a PLB score greater than 0, together with the known binding sites on tubulin of colchicine, taxol and vinca alkaloids.

Site Number	Subunit	Residues lining the site	PLB score
1	A	ARG2 GLU27 HIS28 LYS40 GLY45 ASP46 SER48 PHE49 ASN50 THR51 PHE52 ALA240 LEU242 ARG243 PHE244 ASP245 ASN249 ASN356 GLN358	3.66
2	A/B	GLN134 ASP197 GLU198 TYR200 VAL236 THR237 LEU240 LEU250 LEU253 ALA254 MET257 PHE266 ILE368	2.83
3	A	THR191 HIS192 THR193 THR194 LEU195 GLU196 ARG264 ILE265 PHE267 ASP424 LEU428	2.15
4	A/B	GLN15 THR73 VAL74 ASP76 GLU77 THR80 TYR224 LEU42 ASP45 ARG46 PRO243 GLY244 GLN245 ARG320 MET321 SER322 ASP355	1.68
5	B	ALA19 GLU22 LEU23 LEU26 ARG229 VAL362 VAL363 GLY366 ASP367 LEU368	1.45
6	B	HIS107 THR150 SER151 LEU152 MET154 GLU155 THR193 THR194 GLU196 HIS197 SER198	1.18
7	A	PHE255 ASN258 LEU259 PRO261 MET313 ALA314 CYS315 CYS316 TRP346 CYS347 PRO348 GLY350 PHE351 LYS352	1.10
8	B	ARG2 GLU27 HIS28 LYS40 GLY45 ASP46 SER48 PHE49 ASN50 THR51 PHE52 ALA240 LEU242 ARG243 PHE244 ASP245 ASN249 ASN356 GLN358	0.38
9	A	GLN134 ASP197 GLU198 TYR200 VAL236 THR237 LEU240 LEU250 LEU253 ALA254 MET257 PHE266 ILE368	0.35
10	A/B	THR191 HIS192 THR193 THR194 LEU195 GLU196 ARG264 ILE265 PHE267 ASP424 LEU428	0.22
11	A	GLN15 THR73 VAL74 ASP76 GLU77 THR80 TYR224 LEU42 ASP45 ARG46 PRO243 GLY244 GLN245 ARG320 MET321 SER322 ASP355	0.20
12	A	ALA19 GLU22 LEU23 LEU26 ARG229 VAL362 VAL363 GLY366 ASP367 LEU368	0.06
13	A	HIS107 THR150 SER151 LEU152 MET154 GLU155 THR193 THR194 GLU196 HIS197 SER198	0.04
Colchicine	A/B	αGLU71 αASN101 βARG241 βARG251 βARG318 βALA352	-
Taxol	B	VAL23 ASP26 LEU217 THR219 VAL229 SER230 MET233 PRO272 THR274 SER275 ARG276 GLY277 SER278	-
Vinca	A	ALA247 LEU248 ASN249 LYS352 VAL353	-

Table S7. Predicted GBVI/WSA binding energy of etomidate in each analyzed binding site, reported as mean ± standard deviation among the top 5 poses in each site after energy minimization and rescoring.

Site	GBVI/WSA ΔG [kcal/mol]
1	-6.10 ± 0.18
2	-5.45 ± 0.25
3	-5.22 ± 0.12
4	-5.76 ± 0.13
5	-6.53 ± 0.23
6	-5.51 ± 0.15
7	-5.18 ± 0.04
8	-5.85 ± 0.14
9	-5.34 ± 0.08
10	-6.22 ± 0.29
11	-4.85 ± 0.22
12	-5.29 ± 0.05
13	-5.48 ± 0.21
Colchicine	-5.56 ± 0.23
Taxol	-6.15 ± 0.09
Vinca	-5.90 ± 0.21

Table S8. Predicted GBVI/WSA binding energy of isoflurane in each analyzed binding site, reported as mean ± standard deviation among the top 5 poses in each site after energy minimization and rescoring.

Site	GBVI/WSA ΔG [kcal/mol]
1	-4.21 ± 0.06
2	-4.15 ± 0.16
3	-4.52 ± 0.09
4	-4.27 ± 0.07
5	-4.21 ± 0.15
6	-3.83 ± 0.09
7	-3.93 ± 0.10
8	-3.81 ± 0.18
9	-3.94 ± 0.07
10	-4.19 ± 0.10
11	-3.94 ± 0.07
12	-3.91 ± 0.05
13	-4.21 ± 0.11
Colchicine	-4.22 ± 0.03
Taxol	-4.07 ± 0.10
Vinca	-4.14 ± 0.15

Table S9. Predicted GBVI/WSA binding energy of Picrotoxinin in each analyzed binding site, reported as mean ± standard deviation among the top 5 poses in each site after energy minimization and rescoring.

Site	GBVI/WSA ΔG [kcal/mol]
1	-5.03 ± 0.09
2	-5.26 ± 0.09
3	-4.89 ± 0.03
4	-5.11 ± 0.29
5	-5.54 ± 0.11
6	-5.03 ± 0.12
7	-4.92 ± 0.05
8	-4.82 ± 0.18
9	-4.93 ± 0.07
10	-5.00 ± 0.33
11	-4.60 ± 0.14
12	-4.87 ± 0.15
13	-0.40 ± 1.12
Colchicine	-1.16 ± 0.71
Taxol	-5.57 ± 0.20
Vinca	-0.46 ± 1.04

Table S10. Predicted GBVI/WSA binding energy of picrotin in each analyzed binding site, reported as mean ± standard deviation among the top 5 poses in each site after energy minimization and rescoring.

Site	GBVI/WSA ΔG [kcal/mol]
1	-5.23 ± 0.11
2	-5.3 ± 0.24
3	-5.08 ± 0.09
4	-5.36 ± 0.17
5	-5.55 ± 0.11
6	-4.94 ± 0.15
7	-4.94 ± 0.11
8	-5.03 ± 0.1
9	-4.86 ± 0.29
10	-5.32 ± 0.1
11	-4.46 ± 0.17
12	-4.8 ± 0.3
13	-3.84 ± 0.42
Colchicine	-4.29 ± 1.12
Taxol	-5.58 ± 0.12
Vinca	-3.68 ± 0.58

Table S11. Spectral overlap integrals between tyrosine and tryptophan residues.

**D-A pair**	**J_{SO} (10^{-6} cm)**
Tryptophan > Tryptophan	7.53
Tyrosine > Tyrosine	16.15
Tryptophan > Tyrosine	5.63
Tyrosine > Tryptophan	30.52

Table S12. Magnitudes of hole ($|t_h|$) and electron transfer integrals ($|t_e|$) for TYR dimers at short inter-aromatic distances.

	R_{IA}(Å)	$\|t_h\|$ (cm^{-1})	$\|t_e\|$ (cm^{-1})
**Dimer A**	5.36	49.62	34.83
**Dimer B**	5.02	12.62	85.56
**Dimer C**	4.97	52.25	137.47
**Dimer D**	6.22	51.25	175.40
**Dimer E**	5.18	89.53	419.39

SI References

(1) Bateman, A.; Martin, M. J.; Orchard, S.; Magrane, M.; Agivetova, R.; Ahmad, S.; Alpi, E.; Bowler-Barnett, E. H.; Britto, R.; Bursteinas, B.; et al. UniProt: the universal protein knowledgebase in 2021. *Nucleic Acids Research* **2021**, *49* (D1), D480-D489. DOI: 10.1093/nar/gkaa1100 TubulinDocking2022_refs.enl.
(2) Alushin, G. M.; Lander, G. C.; Kellogg, E. H.; Zhang, R.; Baker, D.; Nogales, E. High-Resolution Microtubule Structures Reveal the Structural Transitions in αβ-Tubulin upon GTP Hydrolysis. *Cell* **2014**, *157* (5), 1117-1129. TubulinDocking2022_refs.enl.
(3) Molecular Operating Environment (MOE) 2019.01. **2019**. TubulinDocking2022_refs.enl.
(4) Labute, P. The generalized Born/volume integral implicit solvent model: Estimation of the free energy of hydration using London dispersion instead of atomic surface area. *Journal of Computational Chemistry* **2008**, *29* (10), 1693-1698. DOI: 10.1002/jcc.20933 TubulinDocking2022_refs.enl.
(5) Studer, G.; Rempfer, C.; Waterhouse, A. M.; Gumienny, R.; Haas, J.; Schwede, T. QMEANDisCo—distance constraints applied on model quality estimation. *Bioinformatics* **2020**, *36* (6), 1765-1771. DOI: 10.1093/bioinformatics/btz828 TubulinDocking2022_refs.enl.
(6) Lindorff-Larsen, K. P., Stefano; Palmo, K.; Maragakis, P.; Klepeis, J. L.; Dror, R. O.; Shaw, D. E. Improved side-chain torsion potentials for the Amber ff99SB protein force field. *Proteins: Structure, Function and Bioinformatics* **2010**, *78* (8), 1950-1958. DOI: 10.1002/prot.22711 TubulinDocking2022_refs.enl.
(7) Wang, J.; Wolf, R. M.; Caldwell, J. W.; Kollman, P. A.; Case, D. A. Development and testing of a general amber force field. *Journal of computational chemistry* **2004**, *25* (9), 1157-1174. DOI: 10.1002/jcc.20035 TubulinDocking2022_refs.enl.
(8) Jakalian, A. J., David B.; Bayly, C. I. Fast, efficient generation of high-quality atomic charges. AM1-BCC model: II. Parameterization and validation. *Journal of Computational Chemistry* **2002**, *23* (16), 1623-1641. DOI: 10.1002/jcc.10128 TubulinDocking2022_refs.enl.
(9) Abraham, M. J.; Murtola, T.; Schulz, R.; Páll, S.; Smith, J. C.; Hess, B.; Lindah, E. Gromacs: High performance molecular simulations through multi-level parallelism from laptops to supercomputers. *SoftwareX* **2015**, *1-2*, 19-25. DOI: 10.1016/j.softx.2015.06.001 TubulinDocking2022_refs.enl.
(10) Bussi, G.; Donadio, D.; Parrinello, M. Canonical sampling through velocity rescaling. *The Journal of chemical physics* **2007**, *126* (1). DOI: 10.1063/1.2408420 TubulinDocking2022_refs.enl.
(11) Berendsen, H. J. C.; Postma, J. P. M.; Van Gunsteren, W. F.; DiNola, A.; Haak, J. R. Molecular dynamics with coupling to an external bath. *Journal of Chemical Physics* **1984**, *81* (8). TubulinDocking2022_refs.enl.
(12) Parrinello, M.; Rahman, A. Polymorphic transitions in single crystals: A new molecular dynamics method. *Journal of Applied Physics* **1981**, *52* (12), 7182-7190. DOI: 10.1063/1.328693 TubulinDocking2022_refs.enl.
(13) Soga, S.; Shirai, H.; Koborv, M.; Hirayama, N. Use of amino acid composition to predict ligand-binding sites. *Journal of Chemical Information and Modeling* **2007**, *47* (2), 400-406. DOI: 10.1021/ci6002202/asset/images/ci6002202.Social.Jpeg_v03 TubulinDocking2022_refs.enl.

(14) Corbeil, C. R.; Williams, C. I.; Labute, P. Variability in docking success rates due to dataset preparation. *Journal of Computer-Aided Molecular Design* **2012**, *26* (6). DOI: 10.1007/s10822-012-9570-1 TubulinDocking2022_refs.enl.
(15) LaFrance, B. J.; Roostalu, J.; Henkin, G.; Greber, B. J.; Zhang, R.; Normanno, D.; McCollum, C. O.; Surrey, T.; Nogales, E. Structural transitions in the GTP cap visualized by cryo-electron microscopy of catalytically inactive microtubules. *Proceedings of the National Academy of Sciences* **2022**, *119* (2), e2114994119. DOI: doi:10.1073/pnas.2114994119.
(16) Pettersen, E. F.; Goddard, T. D.; Huang, C. C.; Meng, E. C.; Couch, G. S.; Croll, T. I.; Morris, J. H.; Ferrin, T. E. UCSF ChimeraX: Structure visualization for researchers, educators, and developers. *Protein Science* **2021**, *30* (1), 70-82. DOI: https://doi.org/10.1002/pro.3943.
(17) Kellogg, E. H.; Hejab, N. M. A.; Howes, S.; Northcote, P.; Miller, J. H.; Díaz, J. F.; Downing, K. H.; Nogales, E. Insights into the Distinct Mechanisms of Action of Taxane and Non-Taxane Microtubule Stabilizers from Cryo-EM Structures. *Journal of Molecular Biology* **2017**, *429* (5), 633-646. DOI: https://doi.org/10.1016/j.jmb.2017.01.001.
(18) *Gaussian 16 Rev. C.01*; Wallingford, CT, 2016. (accessed.
(19) Lakowicz, J. R. *Principles of fluorescence spectroscopy*; Springer, 2006.
(20) Baumeier, B.; Kirkpatrick, J.; Andrienko, D. Density-functional based determination of intermolecular charge transfer properties for large-scale morphologies. *Physical Chemistry Chemical Physics* **2010**, *12* (36), 11103-11113, 10.1039/C002337J. DOI: 10.1039/C002337J.
(21) Js, B. catnip (version 1.9).[software]. 2018.
